# Tumor Extracellular Vesicles lncOSLMT Drives Lung Inflammatory Premetastatic Niche Formation in Osteosarcoma via m^6^A‐Dependent hnRNPA2B1/COX‐2 Axis

**DOI:** 10.1002/advs.202519490

**Published:** 2026-01-21

**Authors:** Hongbo Li, Zehao Guo, Yutong Zou, Jiongfeng Zhang, Jixiang Shi, Xihong Fu, Jian Tu, Hao Yao, Xuanxuan Lu, Lili Wen, Xianbiao Xie

**Affiliations:** ^1^ Department of Musculoskeletal Oncology The First Affiliated Hospital of Sun Yat‐Sen University Guangzhou Guangdong China; ^2^ Guangdong Provincial Key Laboratory of Orthopedics and Traumatology Guangzhou Guangdong China; ^3^ Department of Anesthesiology Sun Yat‐sen University Cancer Center State Key Laboratory of Oncology in South China Collaborative Innovation Center for Cancer Medicine Guangzhou Guangdong China; ^4^ Department of Food Science and Nutrition The Hong Kong Polytechnic University Hong Kong China

**Keywords:** extracellular vesicles, m^6^A modifications, nanoparticles, osteosarcoma, premetastatic niche

## Abstract

Tumor‐derived extracellular vesicles (EVs) play critical roles in premetastatic niche (PMN) formation. Here, we demonstrated that EVs from highly metastatic osteosarcoma cell lines preferentially localized to lung fibroblasts and induce inflammatory PMN formation. High‐throughput profiling of EVs‐derived long noncoding RNAs (lncRNAs) identified lncOSLMT as a candidate enriched in EVs and markedly upregulated in patient tumors and serum, with elevated levels correlating with poor prognosis. Mechanistically, in EVs, lncOSLMT directly bound the RNA‐binding protein hnRNPA2B1. Following internalization by recipient lung fibroblasts, hnRNPA2B1 bound to N^6^‐methyladenosine (m^6^A) ‐modified sites in the 3’UTR of *PTGS2* mRNA, leading to transcript stabilization and increased COX‐2 expression. For therapeutic intervention, we developed lactoferrin‐resveratrol (LF‐RES) nanoparticles, which loaded siRNA against lncOSLMT. Intravenous administration of LF‐RES‐siRNA in mice reduced EVs‐induced lung inflammation and suppressed lung metastasis. Combination with EP2/EP4 antagonists produced enhanced anti‐metastatic effects. These findings establish EVs‐derived lncOSLMT as a key regulator of inflammatory PMN formation in the lung and highlight the potential of LF‐RES‐siRNA nanoparticles as a targeted anti‐metastatic therapy in osteosarcoma.

## Graphical abstract

Tumor‐derived extracellular vesicles program inflammatory lung premetastatic niches through selective delivery of long noncoding RNAs. This work reveals EVs‐associated lncOSLMT as a key driver of lung fibroblast activation via m^6^A‐dependent *PTGS2* stabilization. Targeted silencing of lncOSLMT using lactoferrin‐based nanoparticles effectively suppresses lung inflammation and osteosarcoma metastasis.

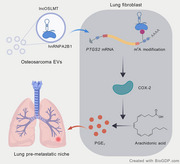

## 1 Introduction

Osteosarcoma, the most prevalent primary malignant bone tumor in children and adolescents, exhibits a highly aggressive clinical course characterized by early metastasis. Despite multimodal therapeutic advances, pulmonary metastasis remains the leading cause of mortality, with 5‐year survival rates plummeting from >70% in localized disease to <20% in metastatic cases [1]. This stark prognosis underscores the urgent need to elucidate the molecular mechanisms driving metastatic dissemination.

Extracellular vesicles (EVs) secreted by tumor cells emerge as pivotal mediators of intercellular communication in the tumor microenvironment. Through the horizontal transfer of bioactive molecules (proteins, lipids, and nucleic acids), EVs contribute to the reprogramming of recipient cells and preparation of distant sites for metastatic seeding [2]. In this context, lncRNAs encapsulated in tumor‐derived EVs have been implicated in educating stromal cells at pre‐metastatic sites [3]. Recent studies have highlighted the functional roles of EVs lncRNAs in premetastatic niche (PMN) formation. For instance, BCSC‐derived exosomal lnc‐PDGFD promoted fibroblast activation in the lungs, fostering a pro‐metastatic microenvironment in triple‐negative breast cancer [4]. Similarly, LINC00482‐enriched EVs contributed to brain metastasis in NSCLC by inducing M2 polarization of microglial cells [5]. In bladder cancer, exosomal lncRNA LNMAT2 promoted lymphangiogenesis by activating PROX1 expression in lymphatic endothelial cells [6]. However, the specific lncRNAs orchestrating PMN formation in osteosarcoma lung metastasis and their mechanistic underpinnings remain largely unexplored.

Herein, we identified lncRNA NR_125944.1, which we termed osteosarcoma lung metastasis associated transcript (lncOSLMT), as a critical driver enriched in osteosarcoma‐derived EVs. lncOSLMT and the RNA‐binding protein hnRNPA2B1 were co‐packaged into EVs via direct interaction. Upon uptake by pulmonary fibroblasts, EVs‐derived hnRNPA2B1 recognized the N^6^‐methyladenosine (m^6^A) site within the 3'UTR of *PTGS2* mRNA, enhancing its stability through an m^6^A‐dependent mechanism. This interaction led to the upregulation of COX‐2 protein and consequent overproduction of prostaglandin E_2_ (PGE_2_), establishing an inflammatory PMN. For therapeutic intervention, we developed a novel nanodrug delivery system consisting of lactoferrin‐resveratrol (LF‐RES) nanoparticles loaded with siRNA targeting lncOSLMT. Our findings provide novel insights into the mechanisms by which osteosarcoma‐derived EVs modulate distant stromal cells and suggest a promising strategy to interfere with EVs‐derived lncRNA‐mediated PMN formation.

## 2 Results

### 2.1 Tumor‐Derived EVs from Highly Metastatic Osteosarcoma Drive Inflammatory PMN Formation in the Lung

**FIGURE 1:**
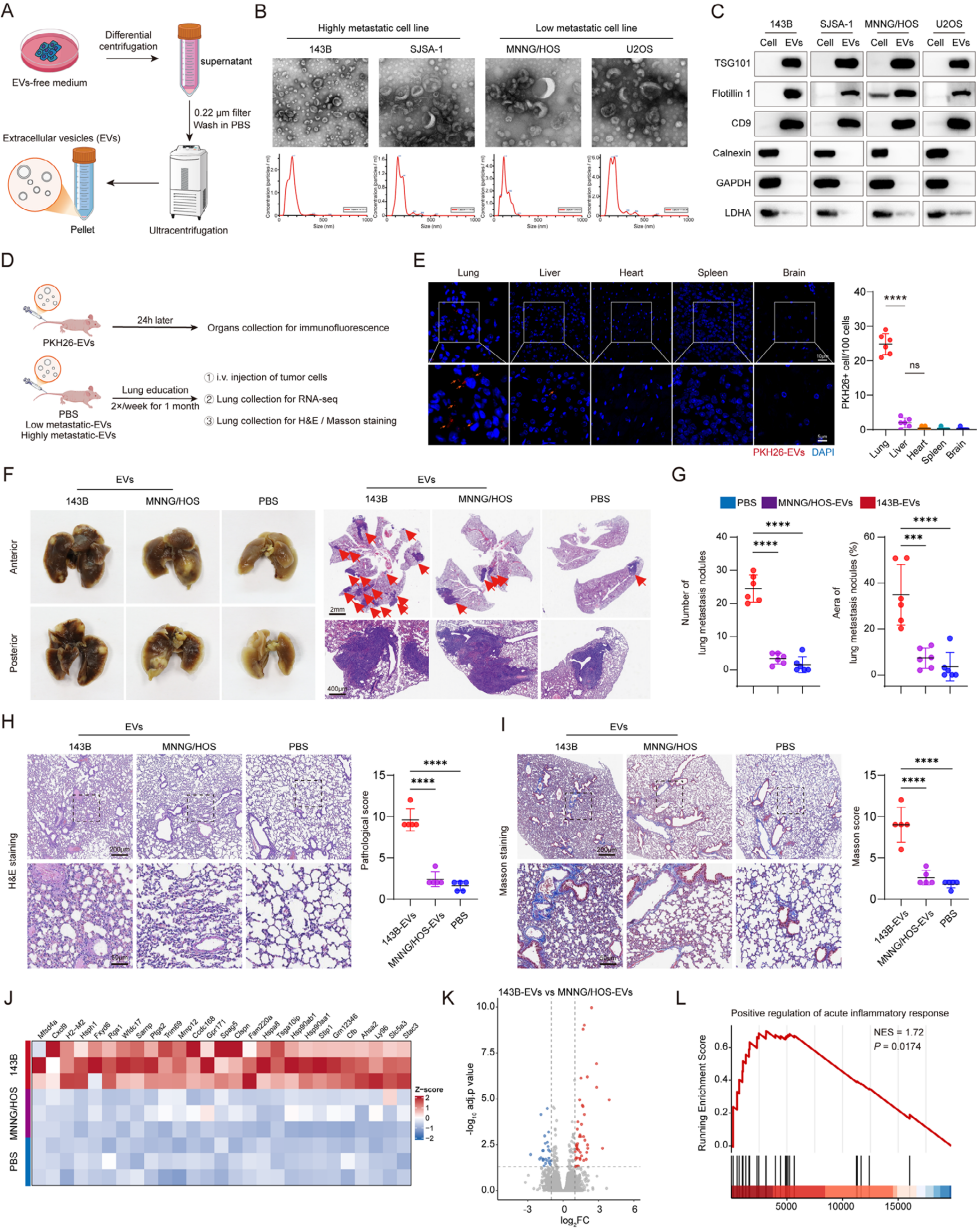
Characterization and functional assessment of EVs in promoting lung metastasis. (A) Schematic illustration of the EV isolation process. (B) TEM image showing the morphology of EVs. (C) Western blot analysis of canonical EVs markers. (D) Schematic diagram of the in vivo experimental design. (E) Representative immunofluorescence images showing the biodistribution of PKH26‐labeled EVs (indicated by orange arrows) in major organs following tail vein injection. Scale bars: 10 µm (upper panels) and 5 µm (lower panels). (F) Gross images and histological sections of lungs from mice pretreated with PBS, MNNG/HOS‐EVs, or 143B‐EVs prior to tumor cell injection. Scale bars: 2 mm (upper panels) and 400 µm (lower panels). (G) Quantification of the number and total area of metastatic nodules in the lungs. (H) Representative H&E staining of lung tissues. Scale bars: 200 µm (upper panels) and 50 µm (lower panels). (I) Representative Masson's trichrome staining of lung tissues. Scale bars: 200 µm (upper panels) and 50 µm (lower panels). (J) Top differentially expressed genes identified from RNA‐seq analysis. (K) Volcano plot showing differentially expressed genes in lung tissues pretreated with 143B‐EVs versus MNNG/HOS‐EVs. (L) GSEA analysis highlighting enrichment of the inflammatory response pathway. Data in (E and G) are presented as mean ± SD (*n* = 6), and data in (H and I) are presented as mean ± SD (*n* = 5). Statistical significance was determined by one‐way ANOVA with appropriate post hoc comparisons. Significance levels are defined as not significant (ns), *p* < 0.001 (^***^), and *p* < 0.0001 (^****^).

EVs were isolated from highly metastatic osteosarcoma cell lines (143B and SJSA‐1) and low metastatic cell lines (MNNG/HOS and U2OS) (Figure 1A; Figure S1A) [7, 8]. The identity was confirmed using TEM (Figure 1B) and western blotting for established EVs markers (Figure 1C; Figure S1B). For the in vivo experiment (Figure 1D), following PKH26 fluorescent labeling and tail vein injection [9], EVs were found to preferentially accumulate in lung tissue (Figure 1E). Consistently, in vivo fluorescence imaging of DiR‐labeled EVs revealed a similar biodistribution pattern (Figure S1C). Subsequently, nude mice were preconditioned via tail vein injection with EVs derived from 143B (highly metastatic) or MNNG/HOS (low metastatic) cells for four weeks, followed by intravenous injection of MNNG/HOS cells. Mice pretreated with 143B‐derived EVs exhibited a significantly higher incidence of lung metastasis compared to PBS and MNNG/HOS EVs groups (Figure 1F,G), indicating that EVs from highly metastatic osteosarcoma cells facilitate tumor colonization in the lungs.

Histological analysis of lung tissues pretreated with 143B‐EVs using H&E (Figure 1H) and Masson's trichrome staining (Figure 1I) revealed prominent inflammatory changes in lung tissues. Consistently, increased protein levels in bronchoalveolar lavage fluid (BALF), together with elevated mRNA expression of *Tnf* and *Il1b* in lung tissues, further supported the occurrence of pulmonary inflammation (Figure S1D). To investigate the underlying molecular alterations, we performed RNA‐seq of lung tissues pretreated with 143B‐EVs versus those treated with MNNG/HOS‐EVs and PBS, which showed a marked upregulation of inflammation‐related genes (Figure 1J,K; Figure S1E–G) and enrichment of the inflammatory signaling pathway (Figure 1L, Figure S1H). These findings demonstrate that EVs from highly metastatic osteosarcoma cells facilitate lung PMN formation through induction of inflammation.

### 2.2 Identification of EVs‐Derived lncOSLMT as a Critical Regulator of PMN Formation and Indicator of Poor Prognosis in Osteosarcoma

**FIGURE 2:**
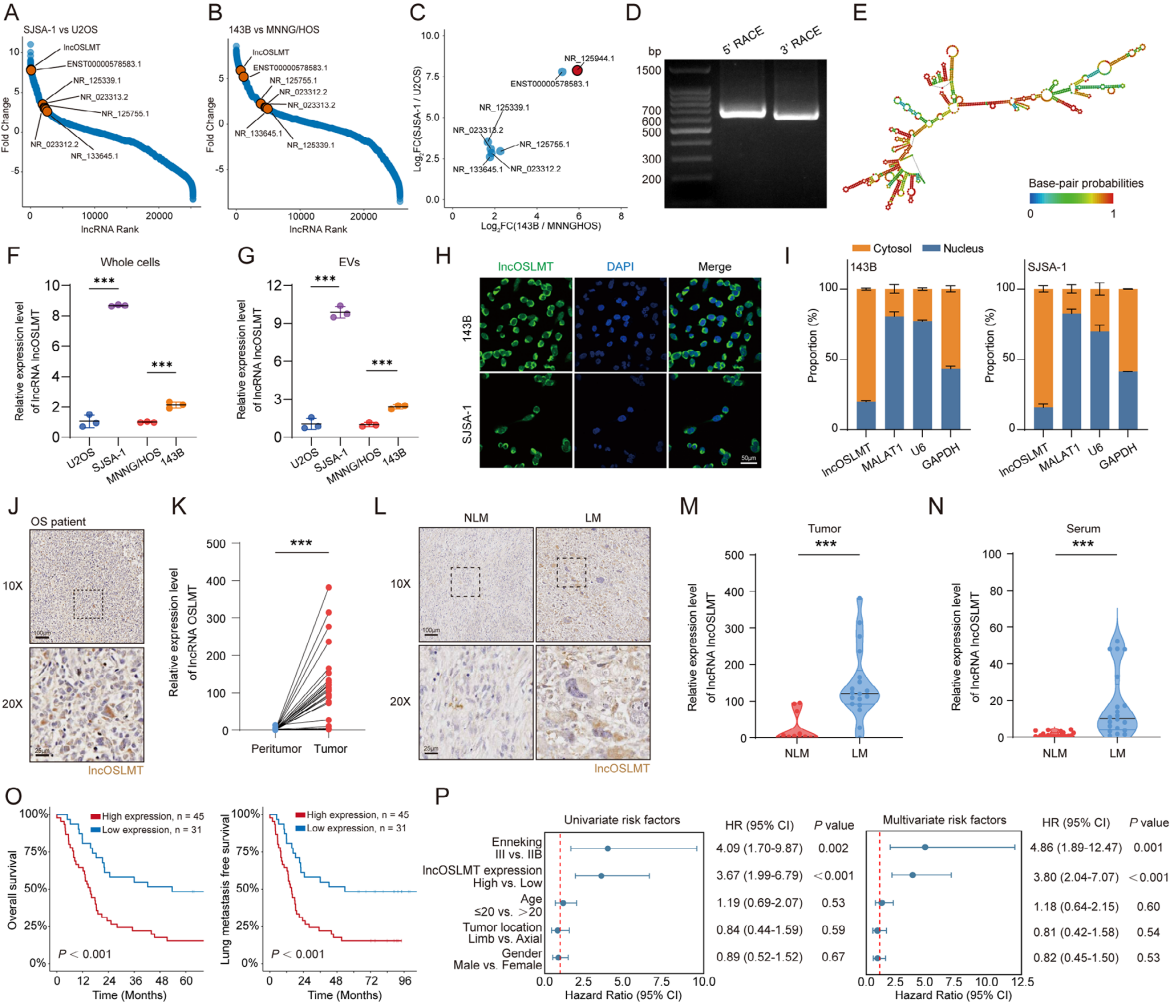
Identification, expression pattern, and clinical relevance of lncOSLMT in osteosarcoma and EVs. (A) Ranked plot of lncRNA expression in SJSA‐1‐derived EVs compared to U2OS‐derived EVs. (B) Ranked plot of lncRNA expression in 143B‐derived EVs compared to MNNG/HOS‐derived EVs. (C) lncOSLMT is the most significantly upregulated lncRNA among commonly elevated candidates (highlighted in red). (D) Agarose gel electrophoresis showing 5’ and 3’ RACE results for lncOSLMT. (E) Predicted secondary structure of lncOSLMT generated by in silico analysis. (F) Relative expression of lncOSLMT in whole cells of different osteosarcoma cell lines (normalized to U2OS). (G) Relative expression of lncOSLMT in EVs derived from osteosarcoma cell lines (normalized to U2OS‐EVs). (H) Representative FISH images showing subcellular localization of lncOSLMT in 143B and SJSA‐1 cells. Scale bars: 50 µm. (I) Nuclear and cytoplasmic fractionation confirmed the subcellular distribution of lncOSLMT in osteosarcoma cells, with lncRNA MALAT1 (nuclear control), *U6* (nuclear control), and *GAPDH* (cytoplasmic control) used as references. (J) ISH in osteosarcoma patient tissues indicates cytoplasmic localization of lncOSLMT. Scale bars: 100 µm (upper panels) and 25 µm (lower panels). (K) Relative expression of lncOSLMT in paired tumor and adjacent normal tissues from osteosarcoma patients. (L) Representative ISH images of lncOSLMT expression in tumors from patients with (LM) or without (NLM) lung metastasis. Scale bars: 100 µm (upper panels) and 25 µm (lower panels). (M) Quantification of lncOSLMT expression in fresh osteosarcoma tissues from patients with or without lung metastasis. (N) lncOSLMT expression levels in serum EVs from osteosarcoma patients with or without lung metastasis. (O) Kaplan‐Meier survival analysis showing the association between lncOSLMT expression and both lung metastasis–free survival and overall survival. (P) Univariate and multivariate analyses of lncOSLMT expression and clinical parameters in osteosarcoma patients. Data in (F,G) are presented as mean ± SD (*n* = 3). Statistical significance was determined using one‐way ANOVA with appropriate post hoc comparisons. Data in (K) are presented as mean ± SD (*n* = 30 pairs). Statistical significance was determined using a paired two‐tailed Student's t‐test. Data in (M) are presented as mean ± SD (*n* = 12 for NLM and *n* = 18 for LM). Statistical significance was determined using an unpaired two‐tailed Student's t‐test. Data in (N) are presented as mean ± SD (*n* = 20 for both NLM and LM). Statistical significance was determined using an unpaired two‐tailed Student's t‐test. Significance levels are defined as *p* < 0.001 (^***^).

Increasing evidence suggests that lncRNAs are enriched in tumor‐derived EVs and contribute to the formation of PMN [10, 11, 12]. In light of these findings, we performed high‐throughput sequencing of EVs‐derived lncRNA from osteosarcoma cell lines with different metastatic potentials (143B vs. MNNG/HOS, and SJSA‐1 vs. U2OS) (Figure 2A,B). Seven lncRNAs (lncOSLMT, ENST00000578583.1, NR_125339.1, NR_023312.2, NR_125755.1, NR_023313.2, NR_133645.1) were consistently enriched in EVs released by highly metastatic osteosarcoma cell lines, among which lncOSLMT exhibited the most significant upregulation (Figure 2C). The full‐length sequence of lncOSLMT was determined by 5′ and 3′ RACE combined with nested PCR (Figure 2D; Figure S2), and its secondary structure was predicted in silicon (Figure 2E). RT‐qPCR confirmed that lncOSLMT was highly expressed both in highly metastatic osteosarcoma cells (Figure 2F) and their derived EVs (Figure 2G). FISH analysis (Figure 2H), nuclear‐cytoplasmic fractionation (Figure 2I) and ISH staining results from patient tissues (Figure 2J) revealed that lncOSLMT predominantly localized in the cytoplasm.

Further expression analysis of osteosarcoma tissues and adjacent tissues revealed significantly higher levels of lncOSLMT in tumor samples (Figure 2K). Importantly, higher lncOSLMT expression was also detected in the tumor tissues (Figure 2L,M) and serum EVs (Figure 2N) of patients with lung metastases. Kaplan‐Meier survival analysis demonstrated that elevated lncOSLMT expression was associated with shorter lung metastasis‐free survival and overall survival (Figure 2O; Table S1). Both univariate and multivariate Cox regression analyses identified high lncOSLMT expression as an independent risk factor for poor prognosis (Figure 2P; Table S2). These suggests that lncOSLMT serves as a potential prognostic biomarker in osteosarcoma.

**FIGURE 3:**
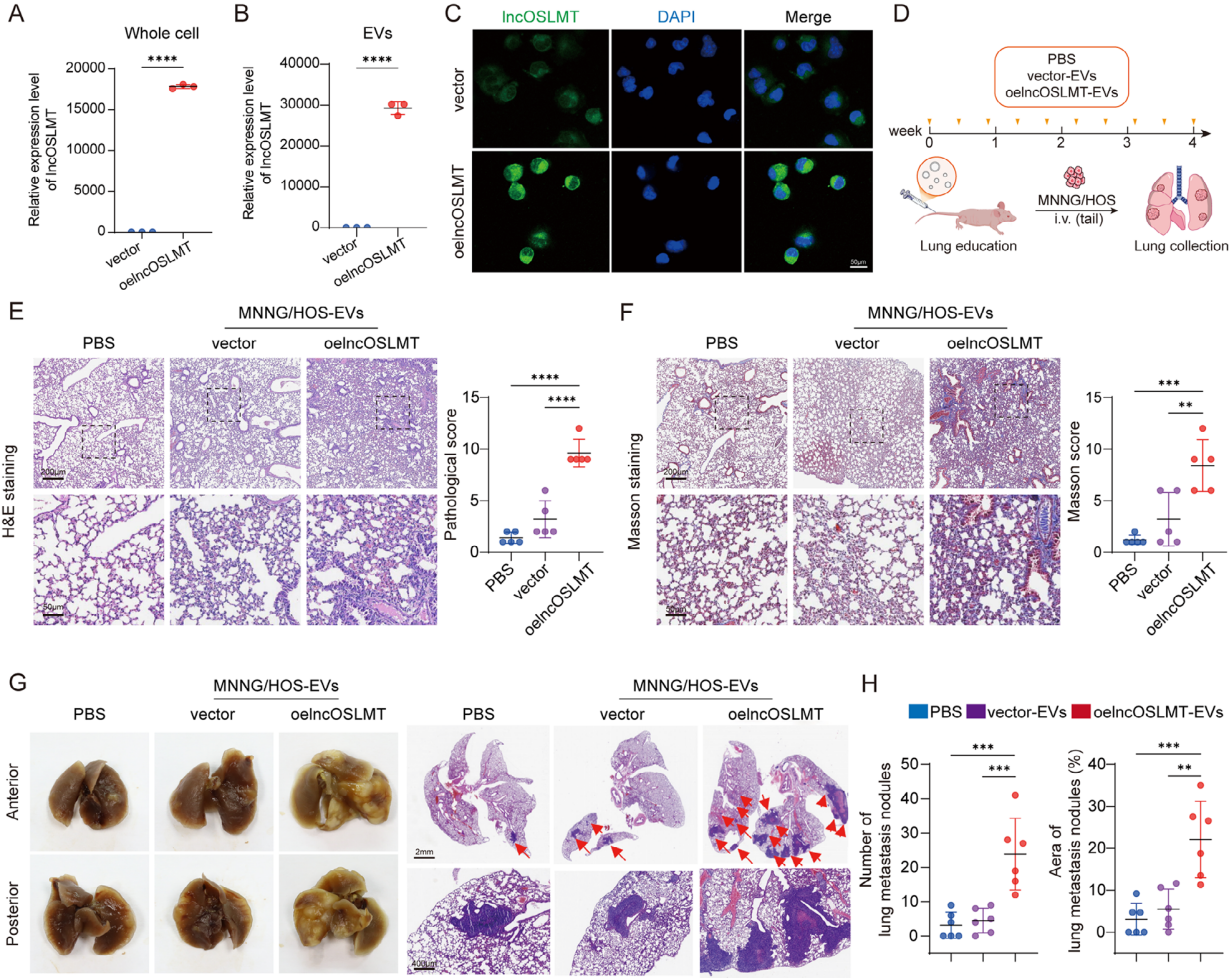
EV‐transferred lncOSLMT promotes lung microenvironment remodeling and metastatic colonization in vivo. (A) Relative expression of lncOSLMT in whole MNNG/HOS cells after transfection with empty vector or lncOSLMT overexpression construct. (B) Relative expression of lncOSLMT in EVs derived from MNNG/HOS cells transfected with empty vector or lncOSLMT overexpression construct. (C) Representative FISH images showing subcellular localization of lncOSLMT in MNNG/HOS cells transfected with vector or lncOSLMT. Scale bars: 50 µm. (D) Schematic diagram of the in vivo experimental workflow. (E) H&E staining of lung tissues pretreated with PBS, vector‐EVs, or lncOSLMT‐overexpressing EVs. Scale bars: 200 µm (upper panels) and 50 µm (lower panels). (F) Masson's trichrome staining of lung tissues pretreated with PBS, vector‐EVs, or lncOSLMT‐overexpressing EVs. Scale bars: 200 µm (upper panels) and 50 µm (lower panels). (G) Gross and histological images of lung metastases following tail vein injection of tumor cells into mice pretreated with PBS or EVs. Scale bars: 2 mm (upper panels) and 400 µm (lower panels). (H) Quantification of metastatic lung nodules and percentage of metastatic area in each group. Data in (A and B) are presented as mean ± SD (*n* = 3) and analyzed using an unpaired two‐tailed Student's t‐test. Data in (E, F, and H) are presented as mean ± SD (*n* = 5 for E and F; *n* = 6 for H) and analyzed using one‐way ANOVA with appropriate post hoc comparisons. Significance levels are defined as *p* < 0.01(^**^), *p* < 0.001 (^***^), and *p* < 0.0001 (^****^).

To investigate its functional role, lncOSLMT was stably overexpressed in the low metastatic MNNG/HOS cells. The upregulation of lncOSLMT was confirmed both in whole cells (Figure 3A) and EVs (Figure 3B). Additionally, FISH was performed to validate its cellular localization (Figure 3C). EVs isolated from these lncOSLMT‐overexpressing cells were then administered to nude mice via tail vein injection (Figure 3D). Mice preconditioned with these EVs developed evident pulmonary inflammation, as demonstrated by H&E staining (Figure 3E) and Masson's trichrome staining (Figure 3F). This was further supported by increased protein levels in BALF and elevated mRNA expression of *Tnf* and *Il1b* in lung tissues, indicating inflammatory activation (Figure S3A). Upon tail vein injection of tumor cells, a significantly increased incidence of lung metastasis was observed (Figure 3G,H). These suggests that lncOSLMT promotes inflammatory PMN formation in the lungs.

### 2.3 EVs‐Derived lncOSLMT Promotes Pro‐inflammatory Activation of Lung Fibroblasts via the COX‐2/PGE_2_ Axis

**FIGURE 4:**
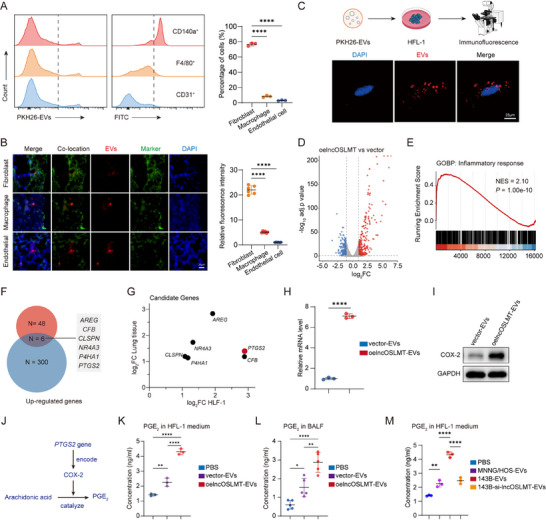
EV‐transferred lncOSLMT activates lung fibroblasts and induces an inflammatory activation via the COX‐2/PGE_2_ axis. (A) Flow cytometric analysis of EV‐positive lung cells following intravenous injection of PKH26‐labeled EVs, showing the proportion of fibroblasts (CD140a^+^), macrophages (F4/80^+^), and endothelial cells (CD31^+^) among EV‐positive cells. (B) Co‐localization of PKH26‐labeled EVs with major lung cell subtypes, including fibroblasts (S100A4^+^), macrophages (F4/80^+^), and endothelial cells (CD31^+^). Scale bars: 25 µm. (C) Schematic diagram of the in vitro EVs uptake experiment and the representative fluorescence images showing the uptake of EVs HFL‐1 cells. Scale bars: 25 µm. (D) Volcano plot of differentially expressed genes in HFL‐1 cells treated with lncOSLMT‐overexpressing EVs versus vector‐EVs. (E) GSEA showing enrichment of the inflammatory response pathway. (F) Venn diagram showing the overlap of upregulated genes between RNA‐seq results from mouse lung tissue and HFL‐1 cells. (G) Log fold changes of selected candidate downstream genes. (H) Quantitative PCR analysis of *PTGS2* mRNA levels in HFL‐1 cells treated with lncOSLMT‐overexpressing or vector‐EVs. (I) Western blot analysis of COX‐2 protein levels in HFL‐1 cells following treatment with lncOSLMT‐overexpressing or vector‐EVs. (J) Schematic illustration of the inflammatory signaling pathway regulated by *PTGS2*. (K) ELISA measurement of PGE_2_ levels in the culture medium of HFL‐1 cells treated with PBS, vector‐EVs, or lncOSLMT‐overexpressing EVs. (L) PGE_2_ concentration in BALF from mice injected with PBS, vector‐EVs, or lncOSLMT‐overexpressing EVs. (M) ELISA measurement of PGE_2_ levels in the culture medium of HFL‐1 cells treated with PBS, MNNG/HOS‐EVs, 143B‐EVs, or 143B‐si‐lncOSLMT‐EVs. Data in (A), (B), (K), (L) and (M) are presented as mean ± SD (*n* = 6 for B, *n* = 3 for A, K, and M, and *n* = 5 for L). Statistical significance was determined using one‐way ANOVA with appropriate post hoc comparisons. Data in (H) are presented as mean ± SD (*n* = 3). Statistical significance was determined using an unpaired two‐tailed Student's *t*‐test. Significance levels are defined as *p* < 0.05 (^*^), *p* < 0.01(^**^), and *p* < 0.0001 (^****^).

We next investigated the cellular localization of the osteosarcoma‐derived EVs within the lung microenvironment. Twenty‐four h after intravenous injection of fluorescently labeled EVs, lung tissues were harvested for cellular uptake analysis. Single‐cell suspensions of lung tissue were analyzed by flow cytometry to quantitatively determine the cellular composition of EV‐positive cells among major resident lung cell populations, including fibroblasts, macrophages, and endothelial cells (Figure 4A). While Frozen lung sections were subjected to immunofluorescence staining to visualize EVS distribution (Figure 4B). Notably, EVs preferentially accumulated in lung fibroblasts. In vitro co‐culture of fibroblasts with EVs confirmed efficient uptake of EVs‐derived cargo (Figure 4C). To further verify that lncOSLMT is transferred via EVs, RAB27a, a known regulator of EV secretion [13], was silenced in 143B tumor cells (Figure S3B), followed by a non‐contact co‐culture with HFL‐1 fibroblasts. Under these conditions, lncOSLMT levels in HFL‐1 cells were markedly reduced (Figure S3C), indicating that EV‐mediated secretion is essential for lncOSLMT transfer.

To explore the specific regulatory effects of lncOSLMT on fibroblasts, RNA‐seq was performed on fibroblasts treated with EVs overexpressing lncOSLMT (Figure 4D). Transcriptomic profiling revealed significant upregulation of the inflammatory response pathway (Figure 4E). Integrated analysis with previous RNA‐seq data from lung tissues treated with highly and low metastatic cell‐derived EVs (Figure 1H) identified six commonly upregulated genes (*PTGS2*, *AREG*, *NR4A3*, *CFB*, *CLSPN*, and *P4HA1*) (Figure 4F), among which *PTGS2* showed the most robust upregulation (Figure 4G), which was previously reported as a key downstream effector of inflammatory response [14, 15]. Consistently, treatment of HFL‐1 cells with EVs derived from highly and low metastatic cells resulted in similar upregulation of these genes (Figure S3D).

Further in vitro validation demonstrated that treatment of fibroblasts with lncOSLMT‐overexpressing EVs significantly increased *PTGS2* mRNA expression (Figure 4H) and COX‐2 protein levels (Figure 4I). COX‐2, encoded by *PTGS2*, is a rate‐limiting enzyme in the synthesis of prostaglandin E2 (PGE_2_), a key mediator involved in inflammation (Figure 4J). Consistently, fibroblasts treated with lncOSLMT‐enriched EVs exhibited elevated PGE_2_ secretion (Figure 4K). In vivo, BALF from nude mice treated with lncOSLMT‐overexpressing EVs also showed increased levels of PGE_2_ (Figure 4L), as PGE_2_ secreted in the lung microenvironment can be directly measured in BALF. Furthermore, ELISA analysis revealed that EVs derived from highly metastatic 143B cells markedly increased PGE_2_ secretion compared with PBS and EVs from low‐metastatic MNNG/HOS cells. Importantly, this effect was significantly attenuated when treated with EVs derived from lncOSLMT‐silenced 143B cells (Figure 4M). These results demonstrate that EVs‐derived lncOSLMT is preferentially taken up by lung fibroblasts, where it induces pro‐inflammatory activation through the COX‐2/PGE_2_ axis.

### 2.4 Coordinated Interaction of lncOSLMT and hnRNPA2B1 in EVs Modulates COX‐2 Signaling in Recipient Cells

**FIGURE 5:**
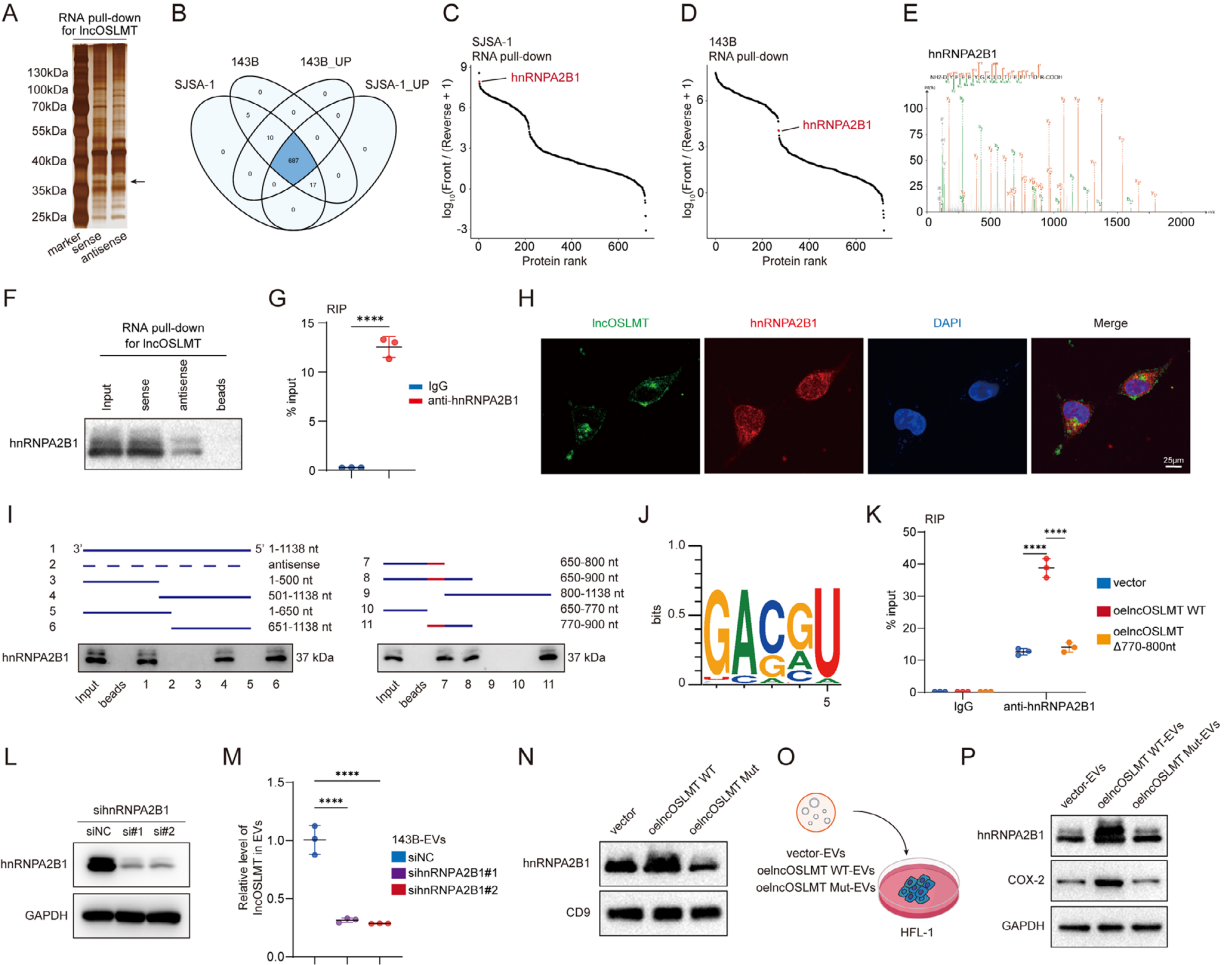
lncOSLMT directly binds hnRNPA2B1 and regulates its activity in EVs‐mediated signaling. (A) Silver staining of proteins pulled down by lncOSLMT. The arrow indicates the differential protein band of interest. (B) Venn diagram showing 687 shared proteins enriched in the sense strand pull‐down compared to the antisense control in both SJSA‐1 and 143B cells (“143B_UP” and “SJSA_UP” indicate proteins enriched in the sense group relative to the antisense control in 143B and SJSA‐1 cells, respectively). (C) Ranked plot of protein abundance (sense vs. antisense) from SJSA‐1 RNA pull‐down. (D) Ranked plot of protein abundance (sense vs. antisense) from 143B RNA pull‐down. (E) Representative MS spectrum of unique peptides corresponding to hnRNPA2B1. (F) RNA pull‐down followed by western blot validation confirms binding between lncOSLMT and hnRNPA2B1. (G) RIP assay verifies the interaction between lncOSLMT and hnRNPA2B1. (H) Representative fluorescence images showing co‐localization of lncOSLMT and hnRNPA2B1 in cells. Scale bars: 25 µm. (I) RNA pull‐down assay using truncated variants of lncOSLMT to map the binding region for hnRNPA2B1. (J) Predicted binding motif of lncOSLMT required for hnRNPA2B1 interaction. (K) RIP assay showing the interaction of hnRNPA2B1 with wild‐type lncOSLMT and a mutant lacking the 770–800 nt binding region. (L) Western blot confirming knockdown efficiency of hnRNPA2B1 at the protein level. (M) Relative levels of lncOSLMT in EVs following hnRNPA2B1 knockdown. (N) Western blot analysis of hnRNPA2B1 protein in EVs derived from cells overexpressing vector, wild‐type lncOSLMT, or lncOSLMT with 770–800 nt mutation. (O) Schematic diagram of EVs treatment experiments in HFL‐1 cells. (P) Protein levels of hnRNPA2B1 and COX‐2 in HFL‐1 cells treated with EVs derived from cells transfected with vector, wild‐type lncOSLMT, or 770–800 nt mutant. Data in (G) are presented as mean ± SD (*n* = 3) and analyzed using an unpaired two‐tailed Student's t‐test. Data in (K,M) are presented as mean ± SD (*n* = 3) and analyzed using one‐way ANOVA with appropriate post hoc comparisons.Significance levels are defined as *p* < 0.0001 (^****^).

RNA pull‐down combined with mass spectrometry identified hnRNPA2B1 (Figure 5A–E), a previously reported RNA‐binding protein (RBP) [16], as a specific interactor of lncOSLMT. The interaction between lncOSLMT and hnRNPA2B1 was further validated by RNA pull‐down (Figure 5F) and RIP (Figure 5G) assays. In addition, FISH analysis demonstrated the subcellular co‐localization of lncOSLMT and hnRNPA2B1 (Figure 5H).

To identify the specific binding region between lncOSLMT and hnRNPA2B1, we constructed a series of truncated RNA fragments (Figure 5I). The analysis revealed that the region spanning nucleotides 770–800 is a potential binding segment (Figure 5J). Deletion of this segment (770‐800 nt) significantly reduced the binding ability of hnRNPA2B1 to lncOSLMT (Figure 5K), indicating this region is critical for the interaction.

We next investigated how this lncRNA‐RBP interaction influences EV packaging and composition. Upon hnRNPA2B1 knockdown (Figure 5L), the level of lncOSLMT in EVs markedly decreased (Figure 5M). Conversely, overexpression of wild‐type lncOSLMT increased hnRNPA2B1 enrichment in EVs, whereas a mutant lacking the 770–800 nt binding region failed to elicit this effect (Figure 5N). These findings suggest that the interaction between lncOSLMT and hnRNPA2B1 influences their incorporation into EVs. EVs derived from cells overexpressing either wild‐type or mutant lncOSLMT were then used to treat fibroblasts (Figure 5O). Western blot analysis revealed that fibroblasts exposed to wild‐type lncOSLMT‐derived EVs exhibited increased hnRNPA2B1 levels and upregulation of the downstream effector COX‐2, whereas mutant lncOSLMT‐EVs had no such effect (Figure 5P). These results suggest that the interaction between lncOSLMT and hnRNPA2B1 is linked to their co‐packaging into EVs and involved in modulating the hnRNPA2B1‐COX‐2 signaling axis in recipient fibroblasts.

### 2.5 hnRNPA2B1 Recognizes m^6^A‐Modified *PTGS2 3*’UTR to Regulate COX‐2 Expression via RNA Stabilization

**FIGURE 6:**
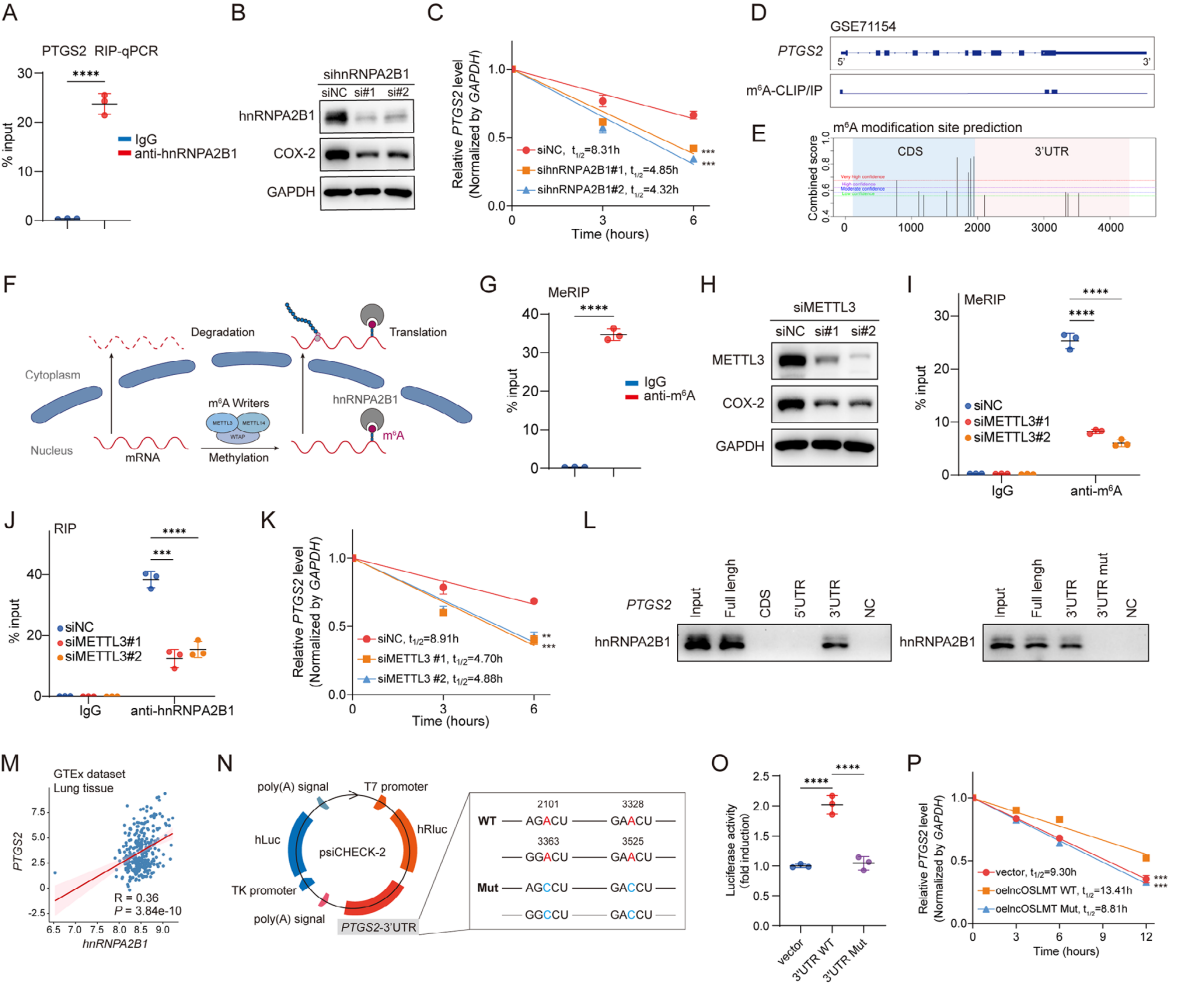
hnRNPA2B1 stabilizes *PTGS2* mRNA via m^6^A‐dependent binding. (A) RIP‐qPCR analysis showing the enrichment of *PTGS2* mRNA by hnRNPA2B1 immunoprecipitation. (B) Western blot analysis of COX‐2 protein levels after hnRNPA2B1 knockdown. (C) *PTGS2* mRNA stability assay following hnRNPA2B1 knockdown. (D) Analysis of GSE71154 dataset indicates the presence of m^6^A modification peaks on *PTGS2* mRNA. (E) SRAMP database prediction of potential m^6^A modification sites on *PTGS2* mRNA. (F) Schematic diagram illustrating that hnRNPA2B1 maintains mRNA stability in an m^6^A‐dependent manner. (G) MeRIP‐qPCR confirms m^6^A modification on *PTGS2* mRNA. (H) Western blot analysis of COX‐2 protein levels following METTL3 knockdown. (I) MeRIP‐qPCR showing decreased m^6^A modification on *PTGS2* mRNA after METTL3 knockdown. (J) RIP‐qPCR showing reduced binding of hnRNPA2B1 to *PTGS2* mRNA after METTL3 knockdown. (K) *PTGS2* mRNA stability assay after METTL3 knockdown. (L) RNA pull‐down using truncated *PTGS2* mRNA fragments to identify hnRNPA2B1 binding regions. (M) Correlation analysis between *PTGS2* and *hnRNPA2B1* mRNA expression levels in lung tissues from the GTEx dataset. (N) Schematic diagram of the dual‐luciferase reporter assay used to assess *PTGS2* mRNA 3’UTR–hnRNPA2B1 interaction. (O) Relative luciferase activity of cells transfected with vector control, *PTGS2* 3'UTR WT, or *PTGS2* 3'UTR Mut reporter plasmids. (P) *PTGS2* mRNA stability assay was performed in HFL‐1 cells treated with EVs overexpressing vector control, wild‐type lncOSLMT, or the Δ770‐800 nt lncOSLMT mutant. Data in (A and G) are presented as mean ± SD (*n* = 3) and analyzed using an unpaired two‐tailed Student's t‐test. Data in (I and O) are presented as mean ± SD (*n* = 3) and analyzed using one‐way ANOVA with appropriate post hoc comparisons. Data in (C and P) are presented as mean ± SD (*n* = 3). Differences among groups across curves were analyzed using two‐way ANOVA. Significance levels are defined as *p* < 0.01(^**^), *p < *0.001 (^***^), and *p* < 0.0001 (^****^).

hnRNPA2B1 has been reported to bind m^6^A‐modified transcripts and regulate their stability [16]. To investigate how hnRNPA2B1 regulates COX‐2 expression, we examined its function as an m^6^A reader and interaction with transcripts. Using RIP‐qPCR, we found that hnRNPA2B1 directly interacts with *PTGS2* mRNA (Figure 6A). Knockdown of hnRNPA2B1 resulted in a marked reduction of COX‐2 protein expression (Figure 6B), accompanied by decreased *PTGS2* mRNA stability (Figure 6C), suggesting a post‐transcriptional regulatory mechanism. Using m^6^A CLIP/IP data from the GEO database (Figure 6D) and the online prediction tool SRAMP (Figure 6E), we identified potential m^6^A modification sites on *PTGS2* mRNA. Based on these findings, we hypothesized that hnRNPA2B1 may bind to the m^6^A‐modified sites on *PTGS2* mRNA to enhance its stability (Figure 6F).

To assess the presence of m^6^A modification on *PTGS2*, we performed m^6^A‐RIP using an anti‐m^6^A antibody and found that *PTGS2* mRNA is significantly enriched (Figure 6G). Notably, knockdown of METTL3 (Figure 6H), a core m^6^A methyltransferase, led to reduced m^6^A levels on *PTGS2* mRNA (Figure 6I), which in turn weakened the binding of hnRNPA2B1 to *PTGS2*, as confirmed by RIP assays (Figure 6J), and further reduced its mRNA stability (Figure 6K). To map the binding region of hnRNPA2B1 on *PTGS2* mRNA, RNA pull‐down assays demonstrated that hnRNPA2B1 primarily binds to the 3’UTR of *PTGS2*. Mutation of the putative m^6^A site within the 3’UTR abolished the interaction between hnRNPA2B1 and *PTGS2* mRNA (Figure 6L), suggesting that this interaction is dependent on m^6^A modification in the 3’UTR. Furthermore, analysis of the RNA‐seq expression data from the GTEx Portal (https://gtexportal.org/) revealed a positive correlation between the mRNA levels of *PTGS2* and *hnRNPA2B1* in lung tissues (Figure 6M). A dual‐luciferase reporter assay further confirmed that the binding of hnRNPA2B1 to *PTGS2* mRNA is mediated through the m^6^A‐modified 3’UTR (Figure 6N,O). Treatment of HFL‐1 cells with EVs carrying wild‐type lncOSLMT significantly increased *PTGS2* mRNA stability, while EVs derived from vector control or Δ770–800 nt lncOSLMT mutant overexpression had no significant impact (Figure 6P). Together, these findings demonstrate that hnRNPA2B1 binds to m^6^A‐modified sites in the *PTGS2* 3’UTR to promote mRNA stability and regulate COX‐2 expression.

### 2.6 LF‐RES‐siRNA Nanoparticles Combine with EP2/EP4 Inhibition to Attenuate Lung Inflammation and Metastasis induced by EVs‐Derived lncOSLMT

**FIGURE 7:**
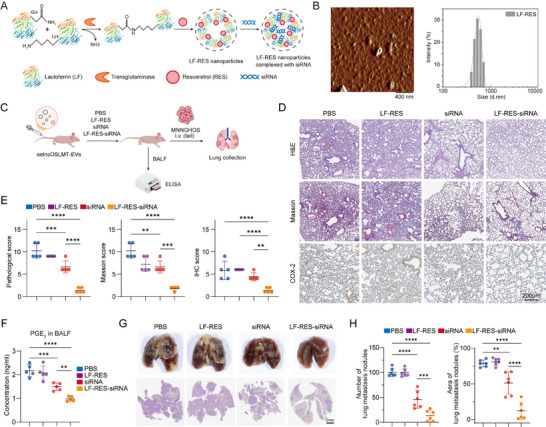
Synthesis, characterization, and therapeutic evaluation of LF‐RES‐siRNA nanoparticles. (A) Schematic illustration of LF‐RES‐siRNA nanoparticle synthesis. (B) AFM morphology and particle size distribution of nanoparticles. (C) Schematic diagram of animal experiments. (D) Representative images of H&E staining, Masson's trichrome staining, and COX‐2 IHC. Scale bars: 200 µm. (E) Quantification of histopathological scores, Masson's trichrome staining scores, and COX‐2 IHC scores. (F) Concentration of PGE_2_ in BALF. (G) Representative gross morphology and histological sections of lung metastases. Scale bars: 2 mm. (H) Quantification of pulmonary metastatic nodule numbers and percentage of lung metastatic area. Data in (E, F, and H) are presented as mean ± SD. Statistical significance was determined using one‐way ANOVA with appropriate post hoc comparisons. Sample sizes were *n* = 5 for (E and F) and *n* = 6 for (H). Significance levels are defined as *p < *0.01(^**^), *p <* 0.001 (^***^), and *p* < 0.0001 (^****^).

The clinical application of siRNA therapy remains limited due to challenges in delivery efficiency, stability, and tissue‐specific targeting [17, 18]. Lactoferrin (LF) is a mammalian cationic glycoprotein with strong tumor‐targeting capabilities via receptor‐mediated endocytosis [19, 20], while resveratrol (RES) is a polyphenolic compound with the ability to enhance nucleic acids transfection [21, 22]. To leverage the synergistic advantages of LF and RES for tumor‐targeted siRNA delivery, LF‐RES nanoparticles were synthesized via a one‐step transglutaminase‐catalyzed crosslinking reaction between glutamine and lysine residues in LF. Subsequently, negatively charged siRNA targeting lncOSLMT was efficiently loaded onto the cationic LF‐RES nanoparticles through simple electrostatic interaction, forming LF‐RES‐siRNA complexes (Figure 7A). The characterization of nanoparticles was performed using AFM (Figure 7B; Figure S4A) and TEM (Figure S4B). The average particle size and zeta‐potential of LF‐RES nanoparticles was 625 ± 58 and 21.30 ± 1.99 mV, respectively. The RES loading content of LF‐RES nanoparticles was 0.18 mg/mL. The *ex vivo* organ biodistribution of the nanoparticles was shown in Figure S4C. Compared with PBS, free siRNA, or LF‐RES alone, LF‐RES‐siRNA nanoparticles significantly alleviated EVs‐induced pulmonary inflammation (Figure 7C,D). This was evidenced by attenuated pathological changes in H&E and Masson‐stained lung sections, decreased COX‐2 expression as shown by IHC (Figure 7E) and reduced PGE_2_ levels in BALF (Figure 7F). Moreover, in a tail vein injection model of tumor metastasis, LF‐RES‐siRNA treatment markedly reduced pulmonary metastatic burden (Figure 7G,H).

**FIGURE 8:**
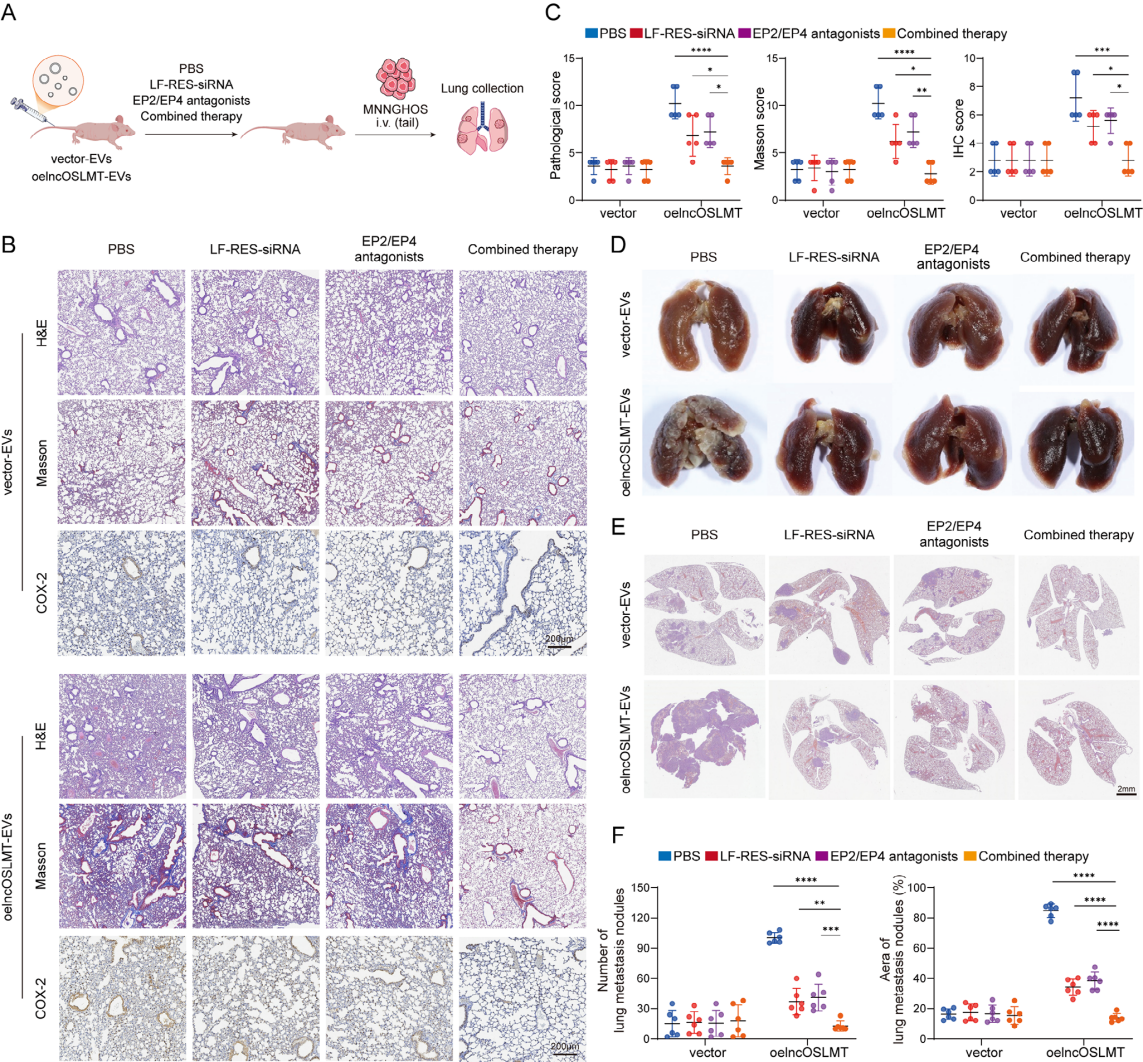
Combined therapeutic effects of LF‐RES‐siRNA nanoparticles and EP2/EP4 antagonists. (A) Schematic diagram of the combined therapy animal experiment. (B) Representative images of H&E staining, Masson's trichrome staining, and COX‐2 IHC after vector‐EVs and oelncRNA‐EVs preconditioning. Scale bars: 200 µm. (C) Quantification of histopathological scores, Masson's trichrome staining scores and COX‐2 IHC scores. (D) Representative gross morphology following EVs preconditioning and tumor cell injection via the tail vein. (E) Representative lung metastatic sections following EVs preconditioning and tumor cell injection via the tail vein. Scale bars: 2 mm. (F) Quantification of pulmonary metastatic nodule numbers and percentage of lung metastatic area. Data in (C and F) are presented as mean ± SD. Statistical significance was determined using one‐way ANOVA with appropriate post hoc comparisons. Sample sizes were *n* = 5 for (C) and *n* = 6 for (F). Significance levels are defined as *p* < 0.05 (^*^), *p* < 0.01(^**^), *p* < 0.001 (^***^), and *p* < 0.0001 (^****^).

Antagonists of the PGE_2_ receptors EP2 and EP4 have been clinically investigated for their anti‐inflammatory and anti‐cancer potential [23, 24]. Interestingly, combining LF‐RES‐siRNA nanoparticles with PGE_2_ receptor, EP2/EP4 antagonists resulted in enhanced therapeutic effects (Figure 8A), significantly outperforming either monotherapy in suppressing inflammation (Figure 8B,C) and reducing metastatic burden in the lung (Figure 8D–F). Our findings demonstrate that LF‐RES‐siRNA nanoparticles provide an efficient siRNA delivery platform. When combined with EP2/EP4 inhibition, this strategy offers a powerful approach to mitigate EVs‐induced lung inflammation and suppress metastasis.

## 3 Discussion

Tumor‑derived EVs play pivotal roles in metastatic progression by preconditioning distant organs and forming PMN [25]. The biodistribution of EVs is directed by molecular cues, leading to a preference for certain target organs. In particular, the organotropism toward lung tissues has been linked to specific protein profiles on EVs and recipient cell surfaces. Exosomal integrins such as α6β4 and α6β1 mediate selective binding and uptake by lung‐resident cells [9]. In parallel, RNAs in EVs were recognized by TLR3 on lung epithelial cells, further enhancing EVs retention in the lung [26]. Our findings demonstrate that EVs derived from highly metastatic osteosarcoma cells are predominantly taken up by lung fibroblasts, leading to pro‐inflammatory activation and formation of an inflammatory PMN, thereby promoting osteosarcoma lung metastasis. lncRNAs transferred via EVs have been shown to mediate organ‐specific metastasis, as reported for breast cancer stem cell‐derived EVs enriched with lnc‐PDGFD promoting lung metastasis and EVs‐derived LINC00482 facilitating lung cancer brain metastasis [4, 5]. In the current study, lncOSLMT is found to be selectively enriched in EVs released by osteosarcoma cells with high metastatic potential. Its elevated expression is positively correlated with the incidence of lung metastasis and serves as an independent prognostic factor for poor outcomes in osteosarcoma patients.

lncRNAs have been reported to exhibit reciprocal interactions with RBPs and function as key regulators of cancer progression through diverse molecular mechanisms. lncRNAs are known to influence the binding capacity of RBPs to their target RNAs, thereby enhancing their post‐transcriptional regulatory functions. Gan et al. reported that FILNC1 inhibited the binding of AUF1 to *c‐Myc* mRNA and suppressed *c‐Myc* expression [27]. In glioblastoma, SChLAP1 has been shown to associate with hnRNP L and potentiated its interaction with *ACTN4* mRNA, leading to increased transcript stability [28]. Here, we demonstrate that the interaction between lncOSLMT and the hnRNPA2B1 is central to a novel EVs‐based regulatory mechanism. lncOSLMT and the RBP hnRNPA2B1 are co‐packaged into EVs via direct interaction. These EVs are preferentially internalized by lung fibroblasts, where hnRNPA2B1 enhances *PTGS2* mRNA expression by stabilizing its m^6^A‐modified transcript. Our findings highlight the essential role of lncRNA‐RBP interaction in linking tumor‐derived EVs signaling to stromal reprogramming.

m^6^A is the most prevalent internal RNA modification in eukaryotes and plays key roles in regulating RNA splicing, translation, and stability. hnRNPA2B1 was first identified as an m^6^A‐reader by Alarcón et al. 2015 [29]. Mechanistically, hnRNPA2B1 recognizes m^6^A motifs in target RNAs via its RNA recognition motifs (RRMs), facilitating selective nuclear export, splicing regulation, or decay. In the context of cell biology, hnRNPA2B1 binds to m^6^A‐modified transcripts and enhances their stability, as demonstrated in multiple models such as *ILF3* mRNA in multiple myeloma and lncRNA NORHA in granulosa cells, regulating proliferation and apoptosis, respectively [30, 31]. In the present study, EVs‐derived hnRNPA2B1 interacts with m^6^A‐modified 3’UTR of *PTGS2* mRNA in lung fibroblasts, increasing the stability of the transcripts, promoting their expression. COX‐2, encoded by *PTGS2*, converts arachidonic acid into PGE_2_, which has been reported to be released from lung‐resident adventitial fibroblasts upon IL‐1β activation to mediate PMN formation [32]. Our data further shows elevated PGE_2_ secretion following EVs stimulation. Histological analysis also confirms the development of pulmonary inflammation, supporting the formation of an inflammatory PMN.

While siRNA‐based therapeutics have made notable clinical progress in recent years, the development of siRNA therapies remains challenging, primarily due to their poor stability in circulation, limited cellular uptake, and lack of targeted delivery [33]. Clinical efforts to silence lncRNAs with siRNA remain scarce, with most studies lacking efficient delivery strategies [34]. These limitations highlight the urgent need for novel delivery platforms. To overcome the limitations of conventional siRNA delivery, we develop a LF‐RES‐based nanoparticle system for targeted silencing of lncOSLMT. LF targets tumors via LF receptors, which are abundantly expressed on various tumor cells [19, 20]. RES, a natural polyphenol, possesses the antioxidant property to enhance while resveratrol (RES) is a polyphenolic compound with the ability to enhance nucleic acids transfection [22, 35]. This system synergistically combines LF‐mediated tumor targeting with the stabilizing effects of RES, enabling efficient delivery of siRNA to tumor and inflamed stromal tissues. LF‐RES‐siRNA nanoparticles effectively reduce COX‐2/PGE_2_‐driven inflammation and suppress lung metastasis in preclinical models. Notably, combination treatment with the EP2/EP4 antagonists yields enhanced effects, suggesting that simultaneous blockade of both upstream lncRNA and key inflammatory mediators represents a promising strategy against metastasis.

## 4 Conclusion

In conclusion, our findings uncover a novel mechanism by which EVs‐derived lncOSLMT facilitates lung metastasis through the regulation of inflammatory PMNs formation. Mechanistically, lncOSLMT binds to hnRNPA2B1, thereby activating lung fibroblasts through the hnRNPA2B1/COX‐2/PGE_2_ axis in an m^6^A modification‐dependent manner. Moreover, we demonstrate the therapeutic potential of LF‐RES‐based siRNA delivery. Combined administration with EP2/EP4 antagonists further improves therapeutic outcomes. These findings offer novel insights into the molecular mechanisms driving organ‐specific metastasis and establish a proof‐of‐concept for lncRNA‐based anti‐metastatic strategies.

## 5 Experimental Section/Methods

### 5.1 Human Samples

Osteosarcoma tissues, matched adjacent tissues and blood samples were obtained from the Department of Musculoskeletal Oncology, The First Affiliated Hospital of Sun Yat‐sen University. All specimens were collected with informed or broad consent from the patients. Study was approved by the Research Medical Ethics Committee of The First Affiliated Hospital of Sun Yat‐sen University.

### 5.2 Animal

BALB/c nude mice (RRID:IMSR_RJ:BALB‐C‐NUDE) aged 4 weeks and weighing 12–16 g were obtained from the GemPharmatech Co. Ltd. and maintained under specific pathogen‐free (SPF) conditions. To precondition the lung microenvironment, mice were intravenously injected with the indicated EVs via the tail vein for 4 weeks. Subsequently, 1 × 10^6^ MNNG/HOS cells suspended in 100 µL PBS were injected into the mice via the tail vein. For nanoparticle therapy, 30 µg of EVs resuspended in 100 µL PBS were administered every other day, together with EP2 antagonist (PF‐04418948, MCE, Cat# HY‐18966, 10 mg/kg), EP4 antagonist (MF498, MCE, Cat# HY‐10794, 10 mg/kg), and 5 nmol LF‐RES‐siRNA, all given every other day for four consecutive weeks. 2 × 10^6^ MNNG/HOS cells suspended in 100 µL PBS were used. Four weeks later, lung metastasis was assessed. All animal experiments were approved by the Institutional Animal Care and Use Committee of Sun Yat‐sen University and conducted in accordance with established guidelines for the care and use of laboratory animals.

### 5.3 Cell Lines

The cell lines used in this study included 143B (RRID: CVCL_2270), SJSA‐1 (RRID: CVCL_1697), U2OS (RRID: CVCL_0042), MNNG/HOS (RRID: CVCL_0439), HFL‐1 (RRID: CVCL_0298) and HEK293T (RRID: CVCL_0063), all of which were obtained from ATCC. All cell lines were cultured in high‐glucose DMEM (Gibco) containing 10% FBS and 1% penicillin/streptomycin (Gibco) at 37°C with 5% CO_2_. All cell lines were authenticated and confirmed to be free of mycoplasma contamination.

### 5.4 Ethics Approval Statement

All animal experiments were approved by the Institutional Animal Care and Use Committee of Sun Yat‐sen University (approval no. [2017]209) and conducted in accordance with established guidelines for the care and use of laboratory animals. Osteosarcoma and blood samples used in this study were approved by the Ethics Committee of The First Affiliated Hospital of Sun Yat‐sen University (approval no. [2021]755). Informed consent was obtained from all patients in the study.

### 5.5 EVs preparation and characterization

EVs were isolated from cell culture medium as follows. Cells were cultured in DMEM supplemented with EVs‐depleted FBS. The collected culture supernatant was sequentially centrifuged at 300 × g for 10 min and 2,000 × g for 10 min at 4°C to remove cells and cell debris. The resulting supernatant was then centrifuged at 10,000 × g for 10 min to eliminate larger vesicles. Subsequently, the supernatant was centrifuged at 10,000 × g for 70 min. The pellet was resuspended in PBS and filtered through a 0.22‐µm pore filter to remove remaining contaminants. The filtrate was ultracentrifuged at 100,000 × g for 70 min at 4°C. The resulting EV pellet was washed with PBS and centrifuged again at 100,000 × g for 70 min. The final pellet, enriched in EVs, was resuspended in PBS for downstream analysis. The morphology of EVs was examined by transmission electron microscopy (TEM). Particle size and concentration were analyzed using the NanoSight LM10 system (Malvern, Framingham, MA) with NTA software (version 3.1). The presence of EV marker proteins was confirmed by Western blotting. EVs were quantified using a BCA assay (Thermo Fisher, Cat# 23227), with 30 µg of EVs resuspended in 100 µL of PBS and administered via tail vein injection.

### 5.6 EVs isolation from patient serum

EVs were isolated from human osteosarcoma patient serum samples using the VEX Exosome Isolation Reagent (from serum) (Vazyme, Cat# R602) following the manufacturer's instructions. Briefly, serum samples were centrifuged at 2,000 × g for 30 min at 4°C to remove cellular debris. The supernatant was carefully transferred to a fresh tube and mixed with the VEX Isolation Reagent at a volume ratio of 5:1 (serum:reagent). After gentle mixing, the samples were incubated at 4°C for 30 min and subsequently centrifuged at 10,000 × g for 10 min. The resulting EVs‐containing pellet was resuspended in nuclease‐free PBS for downstream analyses.

### 5.7 DiR Labeling of EVs and Ex Vivo Organ Imaging

Purified EVs were labeled with a DiR (1,1‐dioctadecyl‐3,3,3,3‐tetramethy‐lindotricarbocyanine iodide) (Invitrogen, Cat# D12731) according to a standard protocol. Labeled EVs were administered via lateral tail vein injection. At defined time points post‐injection, mice were euthanized and perfused with PBS via the right ventricle to remove blood‐borne signal. Organs were excised, gently rinsed in cold PBS, blotted dry, and placed on a black nonreflective imaging tray. *Ex vivo* images were acquired using a PerkinElmer IVIS Lumina III system.

### 5.8 Western Blot

Proteins were extracted using RIPA lysis buffer (Beyotime, China) supplemented with 1 mM phenylmethanesulfonyl fluoride (PMSF). Protein concentrations were measured using the Pierce BCA Protein Assay Kit (Thermo Fisher Scientific, USA). Equal amounts of protein were separated by 10% SDS‐PAGE (EpiZyme, China) and transferred onto PVDF membranes (Millipore, USA). Membranes were blocked with 5% non‐fat milk and incubated overnight at 4°C with appropriate primary antibodies. Subsequently, membranes were incubated with species‐specific HRP‐conjugated secondary antibodies at room temperature for 1 h. Protein bands were visualized using the High‐sig ECL detection reagent (Tanon, China).

### 5.9 RNA Sequencing

Total RNA was extracted from the indicated samples and assessed using the Agilent 2100 Bioanalyzer. mRNA was enriched using oligo(dT) magnetic beads. Library preparation involved RNA fragmentation, synthesis of cDNA, end repair of double‐stranded cDNA, and adaptor ligation, followed by PCR amplification to generate the final sequencing library. After quality control, libraries were sequenced on the BGISEQ‐500 platform. Differential gene expression analysis was performed with the DESeq2 package (RRID: SCR_015687) [36]. Gene Set Enrichment Analysis (GSEA, RRID:SCR_003199) was performed using the GSEA package, with gene sets obtained from the Molecular Signatures Database (MSigDB) (https://www.gsea‐msigdb.org/).

### 5.10 lncRNA Sequencing

Total RNA was extracted from EVs using TRIzol reagent (Invitrogen) following the manufacturer's protocol. The RNA was then fragmented to an average length of approximately 200 bp and used for first‐strand and second‐strand cDNA synthesis, followed by adaptor ligation and library enrichment with low‐cycle PCR, according to the instructions of the NEBNext Ultra RNA Library Prep Kit for Illumina (NEB, USA). The purified libraries were assessed using the Agilent 2200 TapeStation and Qubit 2.0 Fluorometer (Life Technologies, USA). Paired‐end sequencing (PE150, 150 bp read length) was performed on an Illumina HiSeq 3000 platform at Guangzhou RiboBio Co., Ltd.

### 5.11 Dual‐Luciferase Reporter Assay

To assess the post‐transcriptional regulation of *PTGS2* by the 3′UTR, a dual‐luciferase reporter assay was performed using the psiCHECK‐2 vector system (Promega, USA, RRID: Addgene_196655). The wild‐type (WT) and mutant (Mut) 3′UTR sequences of *PTGS2* were amplified by PCR and cloned downstream of the Renilla luciferase gene in the psiCHECK‐2 vector. Putative m^6^A modification sites were predicted using the online tool SRAMP (http://www.cuilab.cn/sramp/). The mutant 3′UTR sequence contained point mutations in the predicted m^6^A sites introduced by site‐directed mutagenesis. After 24–48 h of transfection, luciferase activity was measured using the Dual‐Luciferase Reporter Assay System (Promega, USA) according to the manufacturer's protocol. Briefly, cells were lysed in passive lysis buffer, and the luciferase activity was quantified using a microplate luminometer. Renilla luciferase activity was normalized to firefly luciferase activity, which served as an internal control for transfection efficiency. All experiments were performed in triplicate, and results were presented as mean ± SD.

### 5.12 Histologic Evaluation

For hematoxylin and eosin (H&E) staining, tissues were fixed in formalin, embedded in paraffin, and sectioned at a thickness of 5 µm. The sections were then deparaffinized and stained using an H&E staining kit (Solarbio, G1120‐3) according to the manufacturer's instructions. For Masson's trichrome staining, paraffin‐embedded tissue sections were similarly deparaffinized and rehydrated through a graded alcohol series. The staining was performed using a Masson's trichrome staining kit (Solarbio, G1340) to visualize fibers and nuclei. For immunohistochemical (IHC) staining, sections were deparaffinized and treated with 3% hydrogen peroxide (H_2_O_2_) for 10 min to block endogenous peroxidase activity. Antigen retrieval was carried out using 10 mm citrate buffer (pH 6.0) at high temperature. After cooling, the sections were blocked with 5% bovine serum albumin (BSA) and incubated overnight at 4°C with the primary antibody. The following day, sections were incubated with the appropriate secondary antibody, and staining was visualized using 3,3′‐diaminobenzidine (DAB) substrate. The intensity of IHC staining was independently evaluated by two pathologists, and the degree of immunoreactivity was quantified using the immunoreactive score (IRS) system.

### 5.13 Fluorescence In Situ Hybridization (FISH)

FISH was performed to detect the subcellular localization of the target lncRNA. Briefly, cells were cultured on glass coverslips, washed twice with PBS, and fixed with 4% paraformaldehyde (Solarbio, Cat# P1110) for 15 min at room temperature. After fixation, cells were permeabilized with 0.5% Triton X‐100 (Sigma‐Aldrich, Cat# T9284) for 10 min. Hybridization was carried out using a 5’‐FITC‐labeled oligonucleotide probe specific to the lncRNA. The probe was designed and synthesized by RiboBio (Guangzhou, China). Hybridization was performed in hybridization buffer (Ribo FISH Kit, RiboBio, Cat# C10910) overnight in a humidified chamber. Nuclei were counterstained with DAPI at room temperature. Images were acquired using a confocal fluorescence microscope (ZEISS LSM880).

### 5.14 In Situ Hybridization (ISH)

For tissue‐based detection of the lncRNA, ISH was performed on paraffin‐embedded tissue sections using a digoxigenin (DIG)‐labeled probe. Briefly, 5 µm‐thick sections were deparaffinized in xylene and rehydrated through graded ethanol. Sections were treated with proteinase K (20 µg/mL, Roche, Cat# 03115836001) at 37°C for 30 min, followed by acetylation with 0.25% acetic anhydride in 0.1 M triethanolamine buffer. Hybridization was performed overnight using a DIG‐labeled antisense RNA probe targeting the lncRNA (DIG RNA Labeling Kit, Roche, Cat# 11175025910). DIG signals were detected using anti‐DIG‐AP Fab fragments (Roche, Cat# 11093274910) and visualized with NBT/BCIP substrate solution (Roche, Cat# 11681451001).

### 5.15 RNA Extraction and Quantitative RT‐qPCR

Total RNA was isolated using TRIzol reagent (Invitrogen) following the manufacturer's protocol, and subsequently reverse transcribed into cDNA with the HiScript first Strand cDNA Synthesis Kit (Vazyme, R111). RT‐qPCR was conducted using the Real‐time Fluorescent Quantitative PCR Kit (Vazyme, Q321) on a Fast Real‐Time PCR System (Applied Biosystems, CA, USA). GAPDH served as the internal control, and relative gene expression was determined using the 2^–ΔΔCT method. Primers used were listed in Table S3.

### 5.16 Rapid Amplification of cDNA Ends (RACE) for Full‐Length lncRNA

To obtain the full‐length sequence of the target lncRNA, 5’ and 3’ rapid amplification of cDNA ends (RACE) was performed using the SMARTer RACE 5’/3’ Kit (Takara, USA) according to the manufacturer's instructions. Total RNA was extracted using TRIzol (Invitrogen) and reverse‐transcribed to generate 5’ and 3’ RACE‐ready cDNA. Gene‐specific primers and nested primers were designed based on partial sequences. PCR products were separated by agarose gel electrophoresis.

### 5.17 RNA Pull‐Down

RNA pull‐down assays were carried out using the Pierce Magnetic RNA‐Protein Pull‐Down Kit (Thermo Fisher Scientific, 20164) in accordance with the manufacturer's guidelines. Biotin‐labeled RNAs were synthesized using the T7 Transcription Kit (Thermo Fisher Scientific). For each assay, 50 pmol of biotinylated RNA and 50 µL of magnetic beads were used. Following incubation and three wash steps, the RNA‐bound proteins were analyzed by western blotting, silver staining, or mass spectrometry.

### 5.18 RNA Immunoprecipitation (RIP)

RIP assays were conducted using the Magna RIP RNA‐Binding Protein Immunoprecipitation Kit (17‐700, Millipore) following the manufacturer's protocol. In brief, Protein A/G magnetic beads (Roche, USA) were incubated with 5 µg of specific antibodies and cell lysates from cells overnight at 4°C. After incubation, the resulting immune complexes were washed six times with the provided wash buffer and subsequently treated with proteinase K digestion buffer. RNA was then purified from the complexes, analyzed by qPCR, and normalized to the input control.

### 5.19 MeRIP

MeRIP was conducted as previously published with minor revisions [37]. Total RNA was evaluated with a NanoDrop ND‐1000, and intact mRNA was isolated using an Arraystar Seq‐Star poly(A) mRNA Isolation Kit (Arraystar, MD, USA) according to the manufacturer's instructions. Purified mRNA was randomly fragmented into approximately 100‐nt fragments by incubation in fragmentation buffer. Fragmented mRNA was immunoprecipitated with anti‐m^6^A antibody (Synaptic Systems, Cat# 202003, RRID: AB_2279214), and 1/10 of the fragmented mRNA was kept as input.

### 5.20 RNA Stability Assays

Cells were plated in 6‐well plates and incubated overnight. Cells were then treated with actinomycin D (HY‐17559, MCE) at a final concentration of 5 µg/mL for 0, 3, or 6 h before collection. Total RNA was isolated using TRIzol reagent (Invitrogen, Carlsbad, USA) and analyzed by RT‐PCR. mRNA half‐life was calculated based on previously published methods [38].

### 5.21 Preparation of Lactoferrin‐Resveratrol (LF‐RES) Nanoparticles

Lactoferrin‐resveratrol (LF‐RES) nanoparticles was fabricated using the following method. Briefly, LF was added into 5 mL of water phase (pH 8) at 50 mg/mL and dissolved overnight. RES was dissolved into 0.5 mL of ethanol at 3 mg/mL, which was then mixed with LF solution. The free resveratrol in the mixture was removed by centrifugation at 10,000 × g for 10 min. Transglutaminase (5 mg) was added into the mixture, which was heated at 45°C under stirring for 2 h and then at 80°C for 10 min to inactivate transglutaminase. Finally, the mixture was dialyzed in water for 1 day to obtain LF‐RES nanoparticle suspension, which was stored at 4°C for further use.

### 5.22 Characterization of LF‐RES Nanoparticles

A dynamic light scattering (DLS) instrument (Zetasizer Nano‐ZS, Malvern Instruments, UK) was used to measure the particle size and zeta potential of LF‐RES nanoparticles. An angle of 90° and refractive index of 1.45 were used. The morphology of LF‐RES nanoparticles was characterized using atomic force microscopy (AFM) and transmission electron microscopy (TEM). Sample suspension was dripped onto freshly cleaved mica surface and dried by nitrogen gas. AFM image was obtained by Multimode Nanoscope‐V (Veeco instruments, USA) using tapping mode. A silicon tip with a nominal spring constant of 5N/m was used. LF‐RES nanoparticle suspension was spread on a carbon‐coated copper grid and air‐dried at room temperature. A Tecnai G2 Spirit Twin microscope (FEI, USA) was used to observe the sample under accelerating voltage of 200 kV. The amount of free RE in the dialysis medium was measured using HPLC system, which was used to determine the RE content in LF‐RES nanoparticles by regarding the free RE as unload RE.

### 5.23 Lung Tissue Dissociation and Flow Cytometry

Animals were euthanized by CO_2_ inhalation followed by cervical dislocation. Lungs were excised, placed in cold HBSS, and minced into fragments with sterile scissors. Tissue fragments were transferred into 2 mL digestion buffer with 1 mg/mL Collagenase IV (Worthington), 0.1 mg/mL DNase I (Roche) and 2% FBS and incubated at 37°C for 50 min with gentle agitation. After enzymatic digestion, the tissue was mechanically dissociated by pipetting up and down 10–15 times, and the cell suspension was passed through a 70 µm nylon cell strainer into a 50 mL tube. Cells were centrifuged at 570 × g for 5 min at 4°C, supernatant discarded, and the pellet was resuspended in 2 mL RBC lysis buffer for 2 min to remove residual erythrocytes. Surface staining was performed in a final volume of 100 µL FACS buffer for 30 min at 4°C in the dark with the following antibodies: anti‐mouse CD140a antibody (Thermo Fisher, Cat# 11‐1401‐80, RRID: AB_2572475), anti‐mouse F4/80 antibody (Elabscience, Cat# E‐AB‐F0995C, RRID: AB_3065037), and anti‐mouse CD31 (BioLegend, Cat# 102405, RRID: AB_312900). Data were acquired on a Beckman Coulter CytoFLEX flow cytometer (Beckman Coulter).

### 5.24 Statistical Analysis

All results were derived from at least three independent experiments, and data from one representative experiment are shown. Data are presented as mean ± SD. For comparisons between two groups, either two‐tailed unpaired Student's t test or two‐tailed paired Student's t test was applied as appropriate, depending on the experimental design. Comparisons involving more than two groups were analyzed using one‐way or two‐way analysis of variance (ANOVA), followed by appropriate post hoc multiple‐comparison tests when applicable. Categorical variables were analyzed using the chi‐square test. Survival was assessed with the Kaplan‐Meier method and compared by the log‐rank test. Differences with *p* values < 0.05 were considered statistically significant. All statistical analyses were carried out using R 4.3.1 (www.r‐project.org/) or GraphPad Prism (8.0, GraphPad Software, USA, RRID:SCR_002798).

## Funding

This work was supported by the National Natural Science Foundation of China (Grant Nos. 82303483 and 82273357) and the Guangdong Basic and Applied Basic Research Foundation (Grant No. 2024A1515010075).

## Conflicts of Interest

The authors declare no conflicts of interest.

## Supporting information




**Supporting File**: advs73881‐sup‐0001‐SuppMat.docx.

## Data Availability

The data generated in this study are available upon request from the corresponding author. The RNA sequencing datasets generated in this study have been deposited in the NCBI Gene Expression Omnibus (GEO) under accession numbers GSE308128 and GSE308317, and the lncRNA sequencing dataset has been deposited under accession number GSE308154.
