# Supplementary material for: Tumor Extracellular Vesicles lncOSLMT Drives Lung Inflammatory Premetastatic Niche Formation in Osteosarcoma via m6A‐Dependent hnRNPA2B1/COX‐2 Axis
Source: Adv Sci (Weinh). 2026 Jan 21;13(17):e19490. doi: 10.1002/advs.202519490 (PMC13042700; doi:10.1002/advs.202519490)
Supplement: Supplementary file 1 — Supporting File: advs73881‐sup‐0001‐SuppMat.docx. [file ADVS-13-e19490-s001.docx]

Supporting Information

**Tumor extracellular vesicles lncOSLMT drives lung inflammatory premetastatic niche formation in osteosarcoma via m^6^A-dependent hnRNPA2B1/COX-2 axis**

*Hongbo Li ^#^, Zehao Guo ^#^, Yutong Zou ^#^, Jiongfeng Zhang ^#^, Jixiang Shi, Xihong Fu, Jian Tu, Hao Yao, Xuanxuan Lu*, Lili Wen*, Xianbiao Xie**

This file includes:

Figure S1 to Figure S4, Table S1 to Table S3

**Figure S1. Characterization and functional profiling of osteosarcoma-derived EVs. (A)** Schematic illustration of the extracellular vesicle (EV) isolation and purification workflow. **(B)** Quantitative densitometric analysis of EV marker proteins detected by western blot. Data are presented as mean ± SD (n = 3). Statistical significance was determined using multiple *t*-tests. **(C)** Representative ex vivo fluorescence imaging showing the organ distribution of fluorescently labeled 143B-EVs and HEK-293T-EVs following intravenous injection. **(D)** Total protein concentration in bronchoalveolar lavage fluid (BALF) and qPCR analysis of inflammatory gene expression in lung tissues after EV treatment. BALF total protein content was analyzed with n = 5 mice per group, and lung tissue qPCR analysis was performed with n = 15. Data are presented as mean ± SD. Statistical analysis was conducted using one-way ANOVA with appropriate post hoc comparisons. **(E)** Top differentially expressed genes identified by RNA sequencing in cells treated with 143B-EVs compared with MNNG/HOS-EVs. **(F)** Top differentially expressed genes identified by RNA sequencing in cells treated with 143B-EVs compared with PBS controls. **(G)** Volcano plot showing global transcriptomic changes following 143B-EVs treatment compared with PBS controls, highlighting significantly upregulated and downregulated genes. **(H)** GSEA of RNA-seq data comparing 143B-EVs–treated samples with PBS controls. Significance levels are defined as *P* < 0.01(**), *P* < 0.001 (***), and *P* < 0.0001 (****).


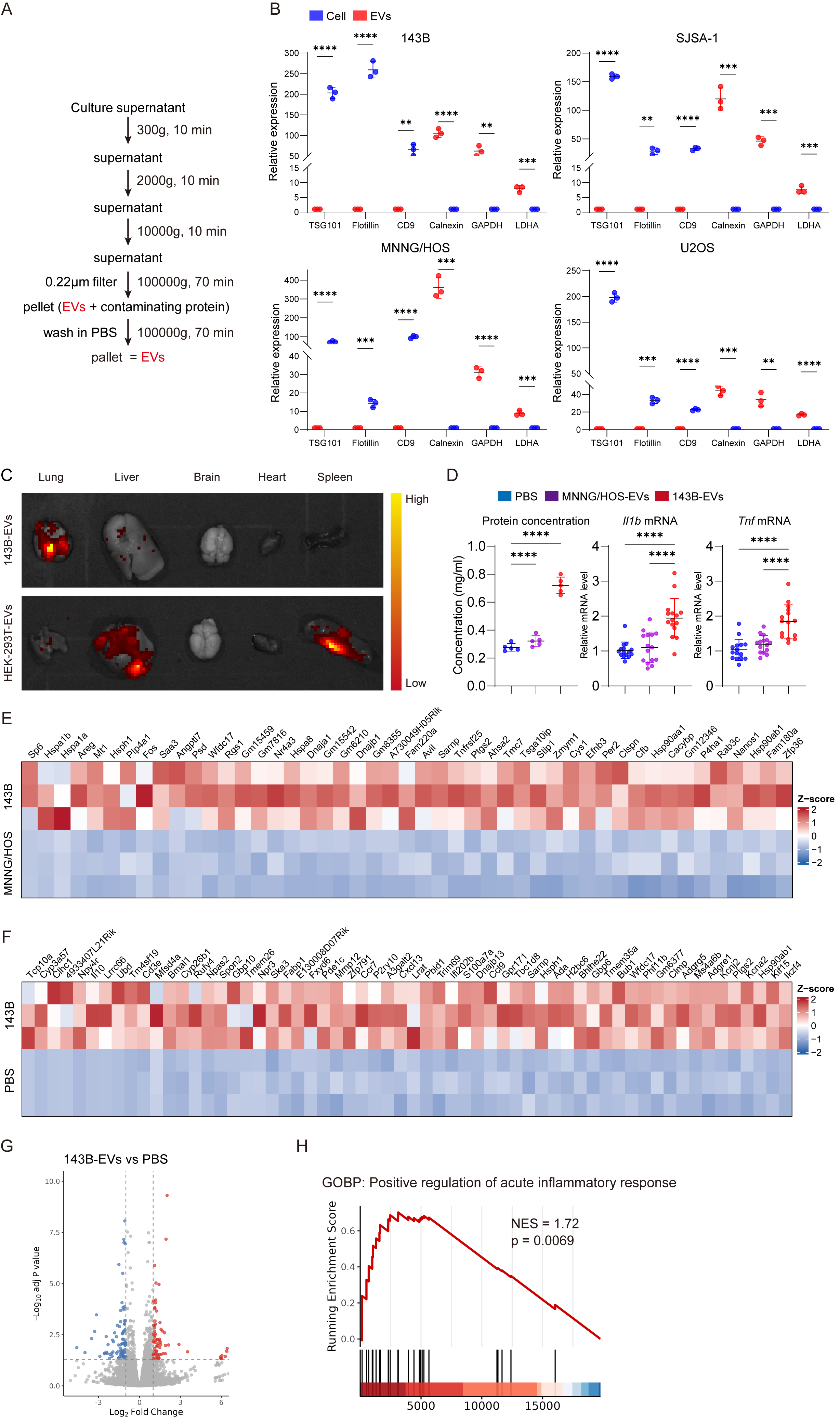


**Figure S2. RACE sequencing results of lncOSLMT.**

**
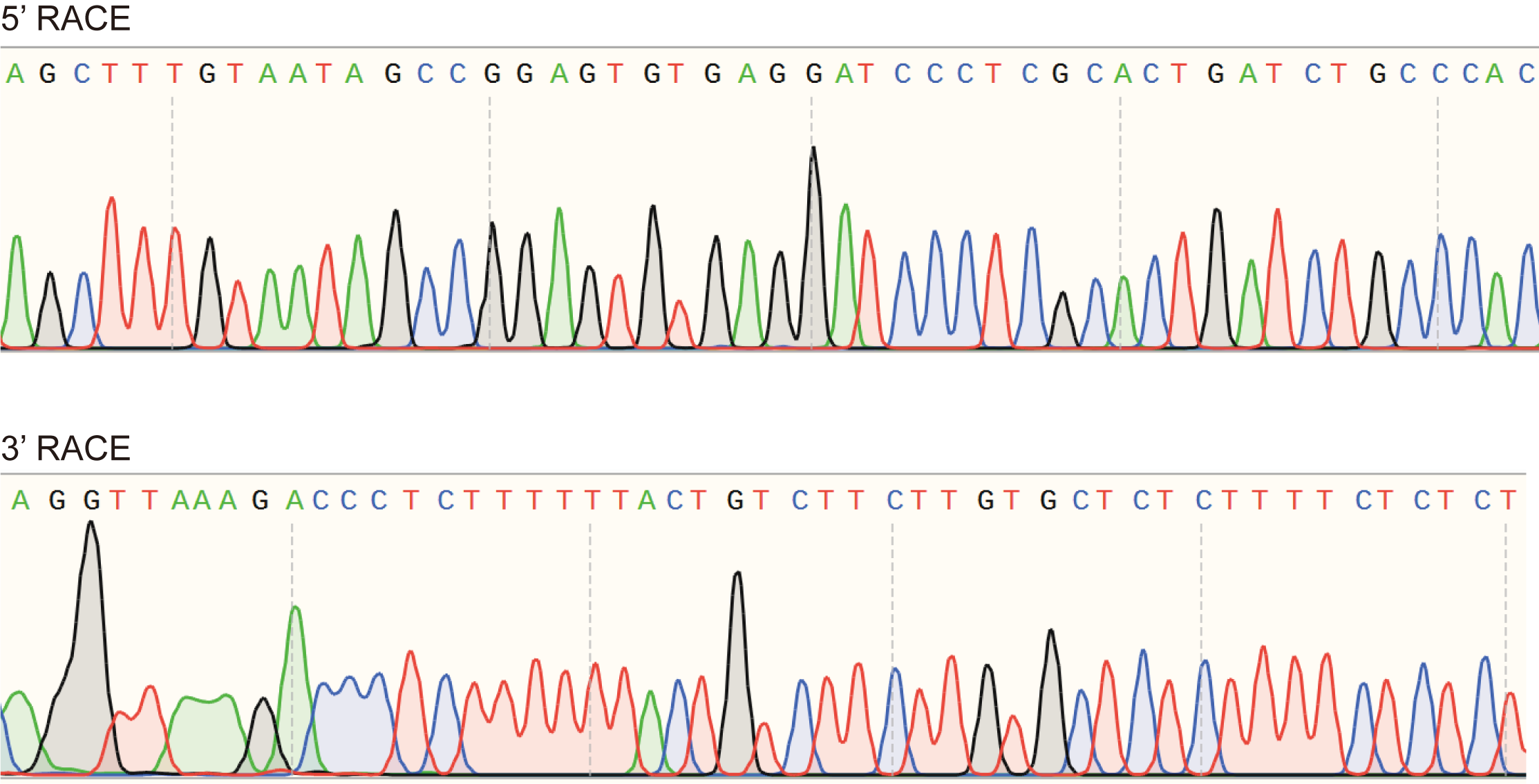
**

**Figure S3. EV-induced pulmonary inflammatory responses and EV-dependent regulation of lncOSLMT. (A)** Total protein concentration in bronchoalveolar lavage fluid (BALF) and qPCR analysis of inflammatory gene expression in lung tissues after EV treatment. BALF total protein content was analyzed with n = 5 mice per group, and lung tissue qPCR analysis was performed with n = 15. Data are presented as mean ± SD. Statistical analysis was conducted using one-way ANOVA with appropriate post hoc comparisons. **(B)** Western blot analysis of RAB27a knockdown efficiency in 143B osteosarcoma cells. **(C)** qPCR analysis of lncOSLMT expression in HFL-1 cells after non-contact co-culture with tumor cells. Data are presented as mean ± SD (n = 3). Statistical significance was determined using an unpaired *t*-test. **(D)** qPCR validation of intersecting genes in HFL-1 cells treated with EVs derived from high- and low-metastatic osteosarcoma cell lines. Data are presented as mean ± SD (n = 3). Statistical significance was determined using multiple *t*-tests. Significance levels are defined as *P* < 0.01(**), *P* < 0.001 (***), and *P* < 0.0001 (****).


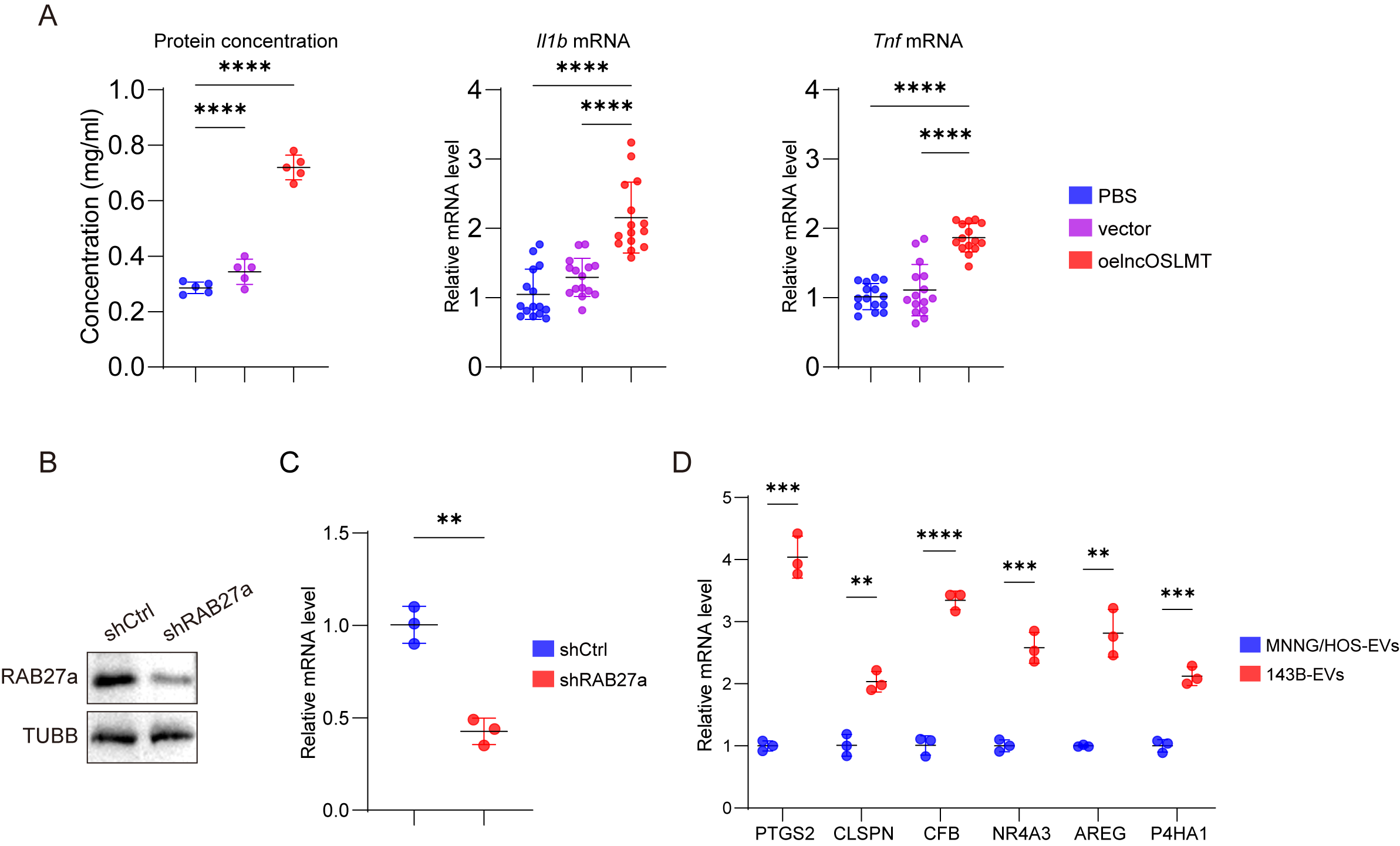


**Figure S4. Physicochemical characterization and in vivo biodistribution of LF-RES-siRNA nanoparticles.**

**(A)** AFM image of LF-RES-siRNA nanoparticles. **(B)** TEM image of LF-RES-siRNA nanoparticles. **(C)** Representative *ex vivo* fluorescence imaging of major organs obtained from mice treated with PBS, LF-RES nanoparticles, free FAM-labeled siRNA, or LF-RES-FAM-siRNA, demonstrating the biodistribution of different formulations.


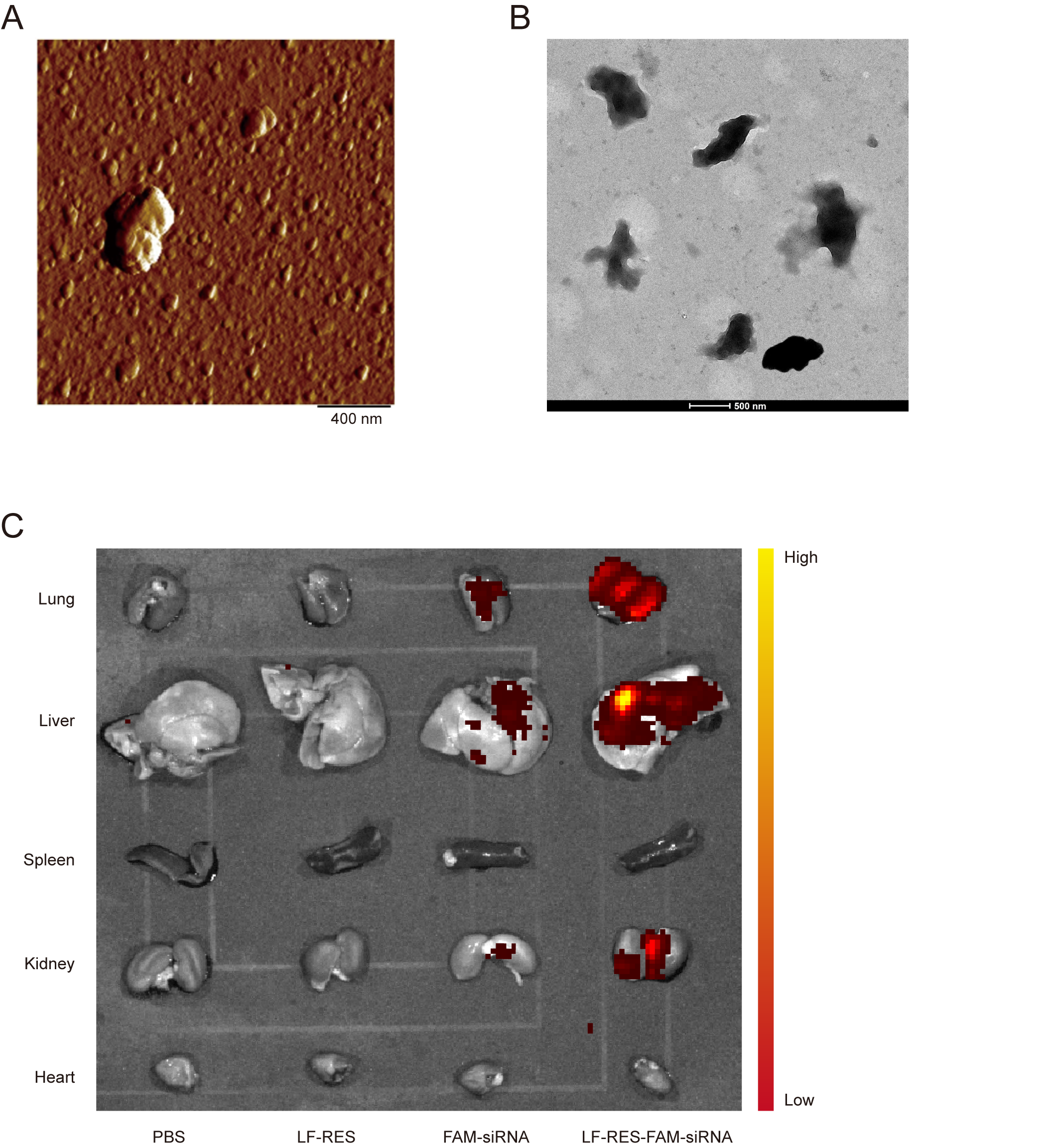


**Table S1. Clinical characteristics of patients included in Kaplan-Meier survival analysis.**

| **Parameters** | **Number of cases** | **lncOSLMT expression** | | ***P* value** |
| --- | --- | --- | --- | --- |
|  |  | **High** | **Low** |  |
| Total | 76 | 45 | 31 |  |
| Age |  |  |  | >0.99 |
| >20 | 51 | 30 | 21 |  |
| ≤20 | 25 | 15 | 10 |  |
| Gender |  |  |  | >0.99 |
| Male | 43 | 25 | 18 |  |
| Female | 33 | 20 | 13 |  |
| Tumor location |  |  |  | >0.99 |
| Limb | 60 | 35 | 25 |  |
| Axial | 16 | 10 | 6 |  |

**Table S2. Clinical characteristics of patients included in univariate and multivariate analyses.**

| **Parameters** | **Number of cases** | **lncOSLMT expression** | | ***P* value** |
| --- | --- | --- | --- | --- |
|  |  | **High** | **Low** |  |
| Total | 82 | 49 | 33 |  |
| Age |  |  |  | >0.99 |
| >20 | 54 | 32 | 22 |  |
| ≤20 | 28 | 17 | 11 |  |
| Gender |  |  |  | >0.99 |
| Male | 49 | 29 | 20 |  |
| Female | 33 | 20 | 13 |  |
| Tumor location |  |  |  | >0.99 |
| Limb | 65 | 38 | 27 |  |
| Axial | 17 | 11 | 6 |  |
| Enneking classification |  |  |  | >0.99 |
| ⅡB | 76 | 45 | 31 |  |
| Ⅲ | 6 | 4 | 2 |  |

**Table S3. Primer sequences used in this study (F: Forward; R: Reverse).**

| **Primer name** | **Sequence (5'-3')** |
| --- | --- |
| lncOSLMT F | TGAGGATCCCTCGCACTGAT |
| lncOSLMT R | GGCTGGGACTTTGGAGTGAA |
| PTGS2 F | ATGCTGACTATGGCTACAAAAGC |
| PTGS2 R | TCGGGCAATCATCAGGCAC |
| GAPDH F | TGCACCACCAACTGCTTAGC |
| GAPDH R | GGCATGGACTGTGGTCATGAG |
| MALAT1 F | GTGCTACACAGAAGTGGATTC |
| MALAT1 R | CCTCAGTCCTAGCTTCATCA |
| U6 F | GCTTCGGCAGCACATATACTAAAAT |
| U6 R | CGCTTCACGAATTTGCGTGTCAT |
| 5’ RACE OUT | AGGTCCAAGAGAGAAAAGAG |
| 5’ RACE IN | GTGTGTTTAGGGAGGTCCAC |
| 5’ RACE INER | TGGTGGAGTGGTCATCAAAC |
| 3’ RACE OUT | TTGATGACCACTCCACCAGA |
| 3’ RACE IN | CAAACGGTATGGCGAGCACT |
| 3’ RACE INER | CCCTGTAAGGGAAGAATCCT |
